# Nonlinear aeroelastic modelling and analysis of a geometrically nonlinear wing with combined unsteady sectional and lifting line aerodynamics

**DOI:** 10.1007/s11071-025-10936-4

**Published:** 2025-02-18

**Authors:** Sanuja Jayatilake, Mark Lowenberg, Benjamin K. S. Woods, Branislav Titurus

**Affiliations:** School of Civil, Aerospace and Design Engineering, University of Bristol, Bristol, UK

**Keywords:** Highly flexible wings, Geometrical nonlinearity, Unsteady aerodynamics, Lifting line method, Limit cycle oscillations, Numerical continuation

## Abstract

This research develops a novel geometrically nonlinear aeroelastic model of a cantilevered flexible wing capable of capturing unsteady aerodynamics with finite span effects. The structural model is developed using a Chebyshev-Ritz approach with the ONERA unsteady aerodynamic formulation coupled to a three-dimensional wing analysis based on the lifting line approach. The resulting system describing the evolution of the generalised structural coordinates and the aerodynamic states is formulated as a continuous state space model. The model is validated against experimental results from the wind tunnel testing of a highly flexible wing demonstrator at the University of Bristol. The numerical results are computed by applying a numerical continuation procedure, exercising the benefits of the state space formulation. The extensive numerical-experimental comparative analysis includes the bifurcation characteristics of the system and the behaviour of the aeroelastic modes. The developed model reflected the experimentally identified bifurcation behaviour. The predicted variations of the flutter onset speed with the wing root pitch angles were found to be within 5% of the experimental values. The model also correctly captured the post-flutter Limit Cycle Oscillation (LCO). This work further showed that the predicted flutter speeds are sensitive to the semi-empirical coefficients present in the ONERA unsteady aerodynamics formulation. Consequently, further calibration of the model was based on the analysis of the experimental flutter speeds and airspeed-driven eigenvalue variations. This approach was necessitated by the influence of application-specific conditions (e.g., the Reynolds number) on the unsteady aerodynamic characteristics. Despite this limitation, the tuned model agreed with the experimental observations across a wide range of test conditions. The approaches presented in this work can benefit multiple applications during research on geometrically nonlinear wings, such as the analysis of root causes influencing critical aeroelastic phenomena or design of aeroelastic control laws.

## Introduction

The study of aircraft with highly flexible wings plays an important role in the development of modern flight vehicles. This is primarily driven by the promise of reduced aerodynamic drag with increasing Aspect Ratio (AR), and the need to achieve high ARs without drowning the aerodynamic benefits with excessive structural weight [1]. Their realisation requires a confident understanding of both static and dynamic aeroelastic behaviours, such as aeroelastic flutter. In the context of increased wing flexibility, these phenomena exhibit highly nonlinear characteristics typically originating from either structural or aerodynamic sources (or both). Particularly with structural geometric nonlinearities, the sensitivity of the aeroelastic modal characteristics on the static equilibrium deflection geometry is significant, ultimately resulting in deflection-dependent flutter instability thresholds [2]. As such, exploring these phenomena requires modelling tools that can confidently capture not only the instability thresholds, but also the underlying aeroelastic modal characteristics. At the same time, the desired features of the utilised model include their ability to capture various physical phenomena (geometrical nonlinearity, unsteady aerodynamics, etc.) whilst being applicable to various analysis methods (time-marching, numerical continuation, eigenvalue problems). This paper develops a new aeroelastic state space model for a geometrically nonlinear cantilevered wing where an unsteady aerodynamic formulation is coupled via a lifting line representation to capture the finite-span effects. The model is validated using an experimental wind tunnel test campaign where comparisons are drawn across both the bifurcation behaviours and against the experimentally derived aeroelastic modal characteristics.

Structural formulations used for aeroelastic simulations can be broadly divided into space-continuous and space-discretised representations. The latter typically utilises finite element approaches [1]. The continuous methods, that are generally seen as low-order modelling approaches, involve the formulations derived using energy-based or variational approaches [3–5]. The resulting structural problems are discretised using either a series of modal coordinates or generalised coordinates corresponding to a series of the shape functions. Whilst often being limited to simpler beam configurations with uniform or smoothly spanwise-varying cross-sections, these models have been extensively used for a range of pioneering classical works [4, 5]. More recent and widely known implementation of this approach includes that by Tang and Dowell [6]. This also includes an extensive experimental comparison against a high AR wing exhibiting flutter. The model utilised there, developed in [3], captured the effects of geometrical nonlinearity through power-series expansions in the structural displacements. This approach was used to approximately express the structural strains in the formulation. Up to second order nonlinear terms in the displacements were retained in the referred work and this was found to be generally sufficient to reflect the behaviour under moderate wing displacements seen in their experiment. A recently developed geometrically exact model [7] that contrasts the one using the power series approximated strains used shape functions to discretise the angular relations and strains in the formulation. Whilst this approach enabled the modelling of arbitrarily large displacements, the description of aerodynamic loads became complex due to the need to establish the appropriate displacements and velocities through the appropriate integral formulations of the strain states. At the same time, a range of the space-discretised structural models, incorporating geometrically exact beam elements, have been developed and applied for modelling highly flexible aircraft. A widely known example is the UM/NAST (University of Michigan’s Nonlinear Aeroelastic Simulation Toolbox) [8]. This uses a strain-based structural formulation [9]. The necessary nodal displacements are obtained by marching the kinematic relationships between strain and displacement along the beam element dimensions. The ASWING modelling tool developed by Drela [10] also employed a spatially discretised structural formulation that was described by a series of nodal displacements and rotations. The system of equations in this case resulted in an (implicit) nonlinear residual form as the governing system of equations includes algebraic constraint equations. Recently, modelling approaches that construct nonlinear models using linear modal bases extracted from finite element models by introducing geometrically nonlinear effects have gained interest [11–13]. For instance, the modal rotation method used in [13], extracts element-wise curvature information from the linear modal basis to derive a nonlinear model by inferring physical displacements and other necessary quantities using nonlinear geometrical relationships. These approaches provide computationally efficient means to model structures with complex geometries (e.g., beams with non-smooth variations of cross-sectional properties).

The aerodynamic formulations used in low to medium order models, such as the ones described above, tend to originate from potential flow-based methods [1]. These approaches can be divided to two groups depending on how the discretisation of the wing is generated for flow analysis. The simpler, and the typically favoured approach is the use of strip theory, coupled with either quasi-steady aerodynamics or a 2D unsteady formulation. The former approach is typically acceptable for cases with very high ARs and lower reduced frequencies, and is favoured for computationally intensive methods of analysis, such as numerical continuation [14]. The second strip theory-based approach benefits from a range of classical and more recently developed 2D unsteady aerodynamic formulations. These range from the Wagner’s and Peter’s models [15] and other semi-empirically derived ones such as the ONERA [16] and Leishman–Beddoes [17] models. The modified ONERA formulation [18] was adopted in the modelling of the high AR demonstrator by Tang and Dowell [6] and was shown to yield a good match with the experiment, including the Limit Cycle Oscillation (LCO) responses. The Peters model was adopted in one application of the UM/NAST model to the Pazy wing demonstrator [19], with synthesised tip-loss factors used to account for the finite-span effects. These approaches, that are well-suited for low order models, have the advantage of being representable in the state space format with the additional aerodynamic states contributing to the system order. In contrast to this, medium order unsteady aerodynamic models, for instance the Unsteady Vortex Lattice Method (UVLM) [20] are generally more computationally intensive and prohibit certain simulation approaches. UVLM aerodynamics has been extensively adopted for aeroelastic analyses in combination with finite element structural models, including SHARPy [21] and the higher order structural MSC NASTRAN model in the DLR formulation [22]. This aerodynamic formulation captures the three-dimensional unsteady wake shedding and its kinematics. However, this process requires convecting the vortex ring elements at each time step and, as a result, the formulation inherently results in a discrete-time state-space system (i.e. the model is described as a map between the specified time steps). The application of this approach to studies beyond the time-marching analysis, if realisable, requires special treatment. For instance, the specialised linearisation technique described in [22] used for stability analysis. Another approach used to capture 3D wake effects employs the Doublet Lattice Method (DLM), used in [23]. However, DLM is only capable of describing aerodynamic loads about an equilibrium at which a linearisation is necessitated. Whilst being a reliable approach for performing flutter computations, this limits its applicability to dynamic responses involving larger deflections where aerodynamic nonlinearity could be exercised. The ASWING formulation [10] employed a method based on the Lifting Line Method [20] to approximate the effects of three-dimensional wake shedding. The unsteady flow physics in this case was approximated by incorporating a single lag term based on the rate of change of circulation in the flow tangency condition. This was combined with a spatially discretised structural formulation, where the combined aeroelastic problem is ultimately described in a nonlinear residual form governing the nodal attributes of the structural elements and the aerodynamic states.

Whilst, generally, the developed low order aeroelastic models are viewed as an inexpensive means for predicting aeroelastic behaviour, more importantly, they also facilitate the interpretation of the experimental observations. The use of numerical models for such tasks stems from the classical work of Goland [5], the more recent work of Tang and Dowell [6], and recently widely discussed Pazy wing demonstrator [24]. These experiments were developed to study the behaviour of aeroelastic flutter instabilities, with the later efforts devoting a greater emphasis to the wing flexibility effects. Aeroelastic flutter typically originates following interactions between different vibratory modes of the system, generally manifesting a two-degree-of-freedom nature due to coupling between a bending and a torsional-dominated mode [25, 26]. This instability may be airspeed bounded (i.e., the system regains stability eventually at a higher speed), as seen in [6] and [24]. The instability in these cases is also referred to as a ‘hump’ mode instability [27] as this originates from a hump-shaped fluctuation of the critically behaved mode’s damping ratio across the negative values with an increasing flow speed. In [6] and [24], wing deflection-dependent migrations of this feature was noted, highlighting the effects of geometrical nonlinearity. The Pazy wing experiment, in particular, was extensively studied against a multitude of modelling frameworks, including the previously mentioned UM/NAST, SHARPy, and the DLR formulations [28]. Across all these numerical comparisons, the emphasis was retained on the aeroelastic bifurcations, from the static aeroelastic responses to the Hopf Bifurcations marking the flutter speeds. This effort, adopting the multitude of models, identified various strengths and drawbacks of different approaches. Notably, research in [19] identified the finite-span effects to significantly influence the hump instability mechanism. This could not be predicted without the finite-span flow physics in the model. Using artificially applied tip-loss factors and lift derivative corrections [29], despite being calibrated to match static responses, yielded varying flutter speeds given the sensitive nature of the hump mode mechanism. This was specifically the case at smaller deflection levels where the approach with the lift derivative corrections showed a largely shrunk hump mode behaviour with a higher flutter onset speed. This finding substantiates the need to compare experimentally measured modal characteristics such as damping ratios with those predicted by models, as these phenomena are ultimately driven by these intricate features.

The modelling approach implemented in this work intends to retain the benefits of a typical low order aeroelastic model, whilst being able to capture the unsteady flow physics with the finite span effects. This research uses the geometrically nonlinear structural model developed and validated by the authors in [30]. This model, that adopts a similar approach to [3], describes two spanwise transversal bending displacements (in-plane and out-of-plane) and a single spanwise torsional displacement of the structure. The overall system is represented in an explicit nonlinear state space form that enables the efficient application of formal nonlinear analysis methods such as numerical continuation. Aiming to capture the finite span effects, the newly proposed aerodynamic modelling approach combines the 2D ONERA formulation with the classical Lifting Line Method [20]. Specifically, this framework combines the capability to model finite wings using the lifting line method, similar to the ASWING framework [10], with the computationally efficient and scalable low order geometrically nonlinear structural formulation whilst benefiting from the explicit state space representation of the problem. The proposed approach is motivated by the lifting line implementation provided in [31] and applied to a rigid wing exhibiting the pitching-heaving motions. This approach differs from the unsteady lifting line implementation described in [10]. Specifically, the strip-wise (‘locally 2D’) unsteady lift coefficients are modelled using a dedicated 2D unsteady aerodynamic model, as opposed to approximating unsteady effects solely by imposing a lag term based on the rate of change of the local bound circulation. In the present scenario, the strip-wise lift coefficients are modelled using only the attached flow equations of the modified ONERA model (used in [6] and [32]). The semi-empirical nature of this locally 2D aerodynamic formulation endows the model with a broad approximation capability. Where sufficient data are available, it allows to capture unsteady aerodynamic characteristics associated with various flow conditions and aerofoils. Furthermore, whilst aerodynamic stall is not considered in the current implementation, the ONERA-based approach retains the possibility of including this feature in future work. The model is comprehensively validated against experimental results from [33].This comprises of a scaled cantilevered wing that exhibits a hump-mode instability, qualitatively similar to the Pazy wing demonstrator behaviour, under conditions where geometrical nonlinearity is exercised. The comprehensive analysis of the experimental results includes indications of the bifurcation behaviour of the system as well as reliable measures of aeroelastic modal characteristics extracted using Operational Modal Analysis.

It is noted that a challenge associated with the model development is that it requires the ONERA coefficients to model the unsteady aerodynamic behaviour specific to the studied flow conditions (e.g., Reynolds number) and the aerofoil. For instance, the unsteady aerodynamic characteristics at lower Reynolds numbers can differ from those modelled using inviscid theory. Given the relatively low Reynolds numbers at which the examined demonstrator was tested and the lack of the established ONERA parameters at these flow conditions, the model required a degree of tuning. In this implementation, the model tuning was achieved by calibration of the ONERA coefficients aimed at matching the predicted and experimentally identified modal frequency-damping characteristics and the flutter onset speeds.

From the extensive comparative analysis using the model with the tuned aerodynamic parameters, it is shown that the model can provide computationally efficient representation of aeroelastic bifurcation behaviours modal characteristics. This includes the migrations of the flutter onset speeds with varying wing root pitch angles, replicated by the model within 5% of the experimental values, and characteristics of fully developed LCO orbits and the evolution of these solution branches from flutter onset. The comparison of the modal characteristics includes the modal frequencies, damping ratios and quantitative measures of the modal coupling developed in [33]. The numerical results used for these comparisons are derived by employing numerical continuation across the same parameter combinations as those implemented in the experiment.

The paper is arranged as follows. Section 2 first introduces the experimentally studied wing demonstrator used for the validation in this paper along with the pertaining test cases from [33]. The newly proposed aeroelastic model is then developed in Sect. 3. Following this, an overview of the wind-off modal characteristics are presented in Sect. 4 along with the setting up of the model to the numerical continuation routines. The central results part of this paper is split in two sections, with Sect. 5 studying the aeroelastic bifurcations and Sect. 6 delving into the modal characteristics.

## Bristol university flexible wing demonstrator

### Experimental demonstrator

**Fig. 1:**
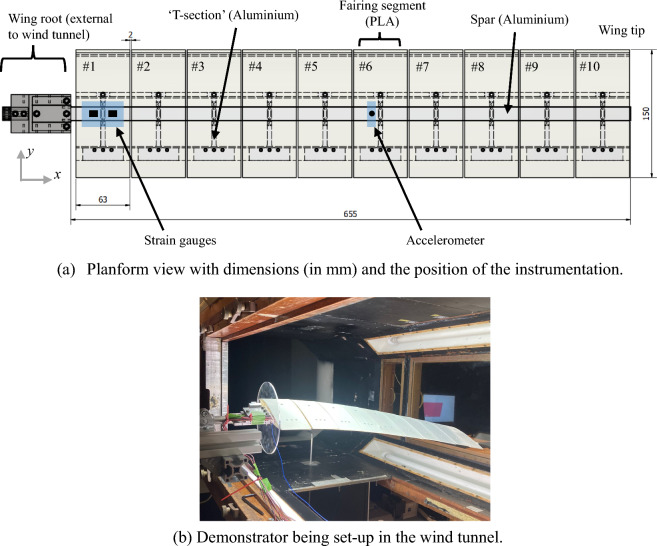
Description of the flexible wing demonstrator

The model-based investigation presented in this paper is centred around the experimentally characterised behaviour of the highly flexible wing demonstrator studied at the University of Bristol. The details of the pertaining experimental procedures and the results are found in [33]. Figure 1 presents the wing demonstrator in the Low Turbulence Wind Tunnel Facility at the University of Bristol along with the internal components and the dimensions. The tests were performed at a range of airspeeds under 30 m/s, with low levels of turbulence (with an intensity of 0.05%).

The cantilevered flexible wing (AR = 8.7) comprised of a uniform aluminium spar, with 10 segmentally attached aerodynamic fairings providing the NACA0018 cross-sectional profile. Each of the 3D printed PLA fairings were attached via a solid T-shaped aluminium section. The spanwise contact length between each interface and the spar was limited to a narrow 5 mm range. This was done to enable each fairing and fairing segment to be modelled as a ‘point-attached’ rigid body located on the spar. Finally, a 2 mm gap included between the adjacent fairing segments ensures that the fairing material does not influence the stiffness properties of the wing across the range of the deflections examined in this work. The physical and geometrical properties of the wing are summarised in Table 1 in the same format later recalled during the model development.

**Table 1:** Physical parameters of the demonstrator

	Description	Value
General dimensions	Beam length, $L$, [m]	0.655
	Semi-chord, $b$, [m]	0.075
Spar	Elastic axis (Theodorsen def.^a^), $a$, [-]	0
	Bending rigidities, $EI_{yy} ,EI_{zz,} EI_{yz}$, [Nm^2^]	2.26, 61.80, 0
	Torsional rigidity, *GJ*, [Nm^2^]	3.30
	Mass distribution, $m$, [kg/m]	0.1242
	Mass centroid, (Theodorsen def.), $e$, [−]	0
Skin + interfaces	Mass, $m_{s}$, [kg]	0.061
	Rotational inertia, $m_{Sxx} ,m_{Syy} ,m_{Szz}$, [kgm^2^]	1.12 × 10^–4^, 1.75 × 10^–5^, 1.12 × 10^–4^
	Mass centroid (Theodorsen def.^a^), $e_{S}$, [–]	0.05
	Lengthwise positions from root, *s,* [m]	$0.0375 + 0.065(j - 1),\;j = 1,\ldots,10$

^a^The elastic axis and the centroid are respectively placed at $b(1 + a)$ and $b(1 + e)$ measured from the leading edge

As indicated in Fig. 1(a), the demonstrator was instrumented with a series of strain gauges near the wing root, and an accelerometer near mid span. The strain gauges were used to extract the wing root bending, torsional and shear loads, as detailed in [33]. These measurements, that provide reliable indicators of the aeroelastic behaviour (e.g. static and dynamic responses including LCO orbits in terms of the wing root loads and accelerations), will be compared with the equivalent predictions obtained from the developed mathematical model. The underlying approach will be further contextualised in Sect. 4.1.

### Overview of test results

The referred experimental results originate from a series of ground and wind tunnel test campaigns. The relevant experimental outcomes from [33] used for the comparisons in the present paper are summarised in Table 2.

**Table 2:** Summary of relevant experimental cases and outcomes from [33]

Test environment	Test method	Outcomes
Ground Vibrations testing (GVT)	Experimental Modal Analysis (EMA) carried out with modal hammer inputs and multiple outputs across the used instruments. Independent variable: Wing root pitch setting	Frequency response functions
		Modal frequencies and damping ratios
		Wind-off mode shapes (‘*reference basis*’) extracted in the form of the sensor participations
Wind tunnel testing	Undisturbed static acquisitions for bifurcation mapping. Independent variables: Wing root pitch setting and the airspeed, *U*	Static wing root load responses at stable equilibria
		Fully developed LCO responses (where encountered)
	Transient, free response acquisitions following external stimulation. Independent variables: Wing root pitch setting and the airspeed, *U*	Frequencies and damping ratios of aeroelastic modes
		Aeroelastic modes, reconstructed using the *reference basis* from GVT (relative participation magnitudes and phase angles between reference modes in each aeroelastic mode)

These experimental outcomes will be used to perform the comparisons with the model developed in the present work. The results from the GVT will be first used to compare the modal frequencies established experimentally against those predicted by the model. These tests were done under the wind-off conditions about various static equilibria induced by varying the angle between the wing root chord and the horizontal direction (‘wing root pitch setting’). This angle was experimentally measured using a digital inclinometer placed on the wing root clamp, illustrated in Fig. 1(a).

The comparisons of the aeroelastic behaviour will be done across two stages whilst varying the airspeed *U*, and the wing root pitch. First, the bifurcation environment of the system, including the stable equilibria, the Hopf Bifurcations and the resulting LCO responses will be assessed. Then, the underlying modal characteristics of the aeroelastic modes will be examined, in particular, by comparing the model-based predictions of the frequencies and damping ratios against the experimental results. This will be complemented with an examination of the modal coupling process using an ad-hoc developed modal projection approach from [33]. All this will be done by numerically investigating the equivalent processes with the mathematical model.

## Mathematical model

### Geometrical definitions

This section introduces the geometrical description of the wing structure. Whilst the structural model developed in [30] is used in this research, the model is fully described here to contextualise its constituents—particularly the handling of geometrical attributes and the application of external forces that are later used to incorporate the aerodynamic-modelling components.

**Fig. 2:**
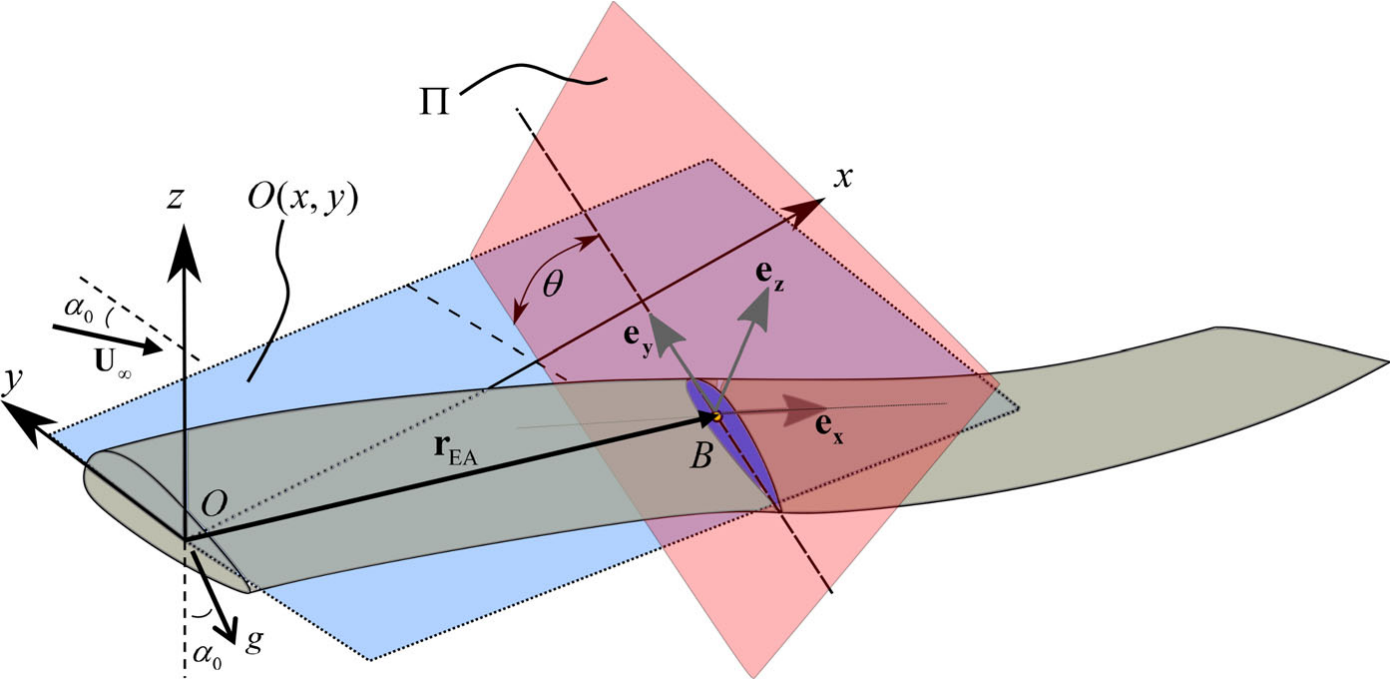
Geometrical definitions of the structural deformations

The geometrical descriptions utilise a fixed frame, *O*, as illustrated in Fig. 2. A secondary frame *B* is considered at each cross-sectional position, of which the orientation will be introduced later. All vectors and displacements, unless explicitly indicated, are expressed in the fixed frame *O*.

*O* is oriented such that the Elastic Axis (EA) of the undeformed wing coincides with the *x* axis, noting the wing in question comprises of a uniform spar with a constant rectangular cross-section. The developed model considers the spatial coordinate, *s*. This is the arc-length along the locus of the EA, and the time coordinate *t*. The model is developed under the assumption of inextensibility of the EA, and as such, *s* uniquely identifies a cross-sectional slice of the wing. The transversal displacement undergone by the EA at *s,* along the *x*, *y* and the *z* axes are, respectively, denoted by *u*(*s,t*)*, v*(*s,t*) and *w*(*s,t*). Hence, the position vector of a point along the EA before deformation,${\mathbf{r}}_{{{\text{EA}}}}^{{{\text{undef}}}} (s)$, and after, ${\mathbf{r}}_{{{\text{EA}}}}^{{}} (s,t)$, can be written as,

1 $$ {\mathbf{r}}_{{{\text{EA}}}}^{{{\text{undef}}}} = \left[ {\begin{array}{*{20}c} s & 0 & 0 \\ \end{array} } \right]^{T} ,\quad {\mathbf{r}}_{{{\text{EA}}}} = \left[ {\begin{array}{*{20}c} {s + u(s,t)} & {v(s,t)} & {w(s,t)} \\ \end{array} } \right]^{T} $$

The inextensibility assumption described above is a result of the significantly higher stiffness of the structure under axial strain such that the significantly softer bending and torsion dominate the dynamics of interest. Means by which this requirement is enforced predominantly include the use of Lagrangian multipliers, after explicitly describing all the translational degrees of freedom (e.g. [34]), or by implicitly expressing one of the translational deformations using the others, based on the inextensibility constraint (e.g. [35]). In this work, the latter approach is favoured given its simplicity.

The inextensibility condition can be mathematically described as $\left| {{\mathbf{r^{\prime}}}_{{{\text{EA}}}} } \right| = 1$, where $\left( \bullet \right)^{\prime }$ is the derivative with respect to the spatial coordinate. The expanded form of this can be written as,

2 $$ \left| {{\mathbf{r^{\prime}}}_{{{\text{EA}}}} } \right|^{2} = (1 + u^{\prime})^{2} + w^{{\prime}{2}} + v^{{\prime}{2}} = 1 $$

The *u* displacement is selected as the one to be described implicitly in this model, with the remaining bending-driven *w* and *v* displacements being calculated explicitly. This is done by adopting the Taylor expansion of the above expression to establish $u^{\prime}$ as the subject of calculations,

3 $$ u^{\prime}(s,t) \approx - \frac{1}{2}\left( {w^{{\prime}{2}} + v^{{\prime}{2}} } \right) - \frac{1}{8}\left( {w^{{\prime}{4}} + v^{{\prime}{4}} + 2w^{{\prime}{2}} v^{{\prime}{2}} } \right) $$

and then integrating along the spatial domain considering the boundary condition $u(0,t) = 0$,

4 $$ u(s,t) \approx - \int\limits_{0}^{s} {\frac{1}{2}\left( {w^{{\prime}{2}} + v^{{\prime}{2}} } \right) + \frac{1}{8}\left( {w^{{\prime}{4}} + v^{{\prime}{4}} + 2w^{{\prime}{2}} v^{{\prime}{2}} } \right)d\overline{s}} $$

To this end, the translational deformations locating the locus of the EA are defined based on the two explicitly described displacements.

Prior to developing the description of a general point of a cross-section, the final geometrical assumptions are outlined: The points along a given cross section at *s* are assumed to always remain in the same plane $\Pi$ indicated in Fig. 2 (i.e., no warping assumption). Plane $\Pi$ is assumed to be perpendicular to the local tangent to the locus of the EA (i.e., perpendicular to ${\mathbf{r^{\prime}}}_{{{\text{EA}}}}$). Each cross-section is hence treated as a rigid planar entity whose position is partially defined by the displacement of the EA attached to the cross-section and its orientation such that it is coplanar to $\Pi$.

The body frame $B$ is attached to the ‘rigid’ cross section where its *x* axis coincides with the local tangent to the EA locus such that ${\mathbf{e}}_{{\mathbf{x}}} = {\mathbf{r^{\prime}}}_{{{\text{EA}}}} /\left| {{\mathbf{r^{\prime}}}_{{{\text{EA}}}} } \right|$. The remainder of this orthogonal triad spans $\Pi$. The final explicitly described torsional deformation, $\theta (s,t)$, defines the orientation of ${\mathbf{e}}_{{\mathbf{y}}}$ and ${\mathbf{e}}_{{\mathbf{z}}}$ in $\Pi$. This angle describes the acute angle between the line of the intersection between $\Pi$ and the $O(x,y)$ plane (i.e., $O(x,y) \cap \Pi$) and the axis ${\mathbf{e}}_{{\mathbf{y}}}$ as illustrated in the figure. The ${\mathbf{e}}_{{\mathbf{z}}}$ axis follows naturally from the orthogonality condition ${\mathbf{e}}_{{\mathbf{z}}} = {\mathbf{e}}_{{\mathbf{x}}} \times {\mathbf{e}}_{{\mathbf{y}}}$. The implicit relationships between the axes ${\mathbf{e}}_{{\mathbf{z}}} ,{\mathbf{e}}_{{\mathbf{x}}} ,{\mathbf{e}}_{{\mathbf{y}}}$ in terms of the three deformations $w(s,t),v(s,t),\theta (s,t)$ are derived in “Appendix A”.

Ultimately, the position vector of a generic point before deformation, ${\mathbf{r}}^{{{\text{undef}}}}$, and after, ${\mathbf{r}}$, can be written as,

5 $$ {\mathbf{r}}^{{{\text{undef}}}} = \left[ {\begin{array}{*{20}c} s & {n_{y} } & {n_{z} } \\ \end{array} } \right]^{{\text{T}}} ,\quad {\mathbf{r}} = {\mathbf{r}}_{{{\text{EA}}}} (s,t) + n_{y} {\mathbf{e}}_{{\mathbf{y}}} (s,t) + n_{z} {\mathbf{e}}_{{\mathbf{z}}} (s,t) $$

where $n_{y}$ and $n_{z}$ are respectively the offsets in the local cross-section along the ${\mathbf{e}}_{{\mathbf{y}}}$ and ${\mathbf{e}}_{{\mathbf{z}}}$ axes.

### Discretisation and functional basis

The adopted modelling approach in this work discretises the previously introduced continuum problem characterised by the two translational, $w(s,t),v(s,t)$, and one torsional, $\theta (s,t)$, displacement. This is done using the Chebyshev-Ritz approach [36, 37], where the spatial and the temporal dependency of each displacement is expressed as the product between the *s*-dependent shape functions and the corresponding time dependent generalised coordinates. In this way, a suitable set of the basis shape functions is used to discretise the continuous functions.

Accordingly, the three displacements are described as,

6 $$ w(s,t) = {\mathbf{Y}}_{w} (s)^{{\text{T}}} {\mathbf{q}}_{w} (t),\quad \theta (s,t) = {\mathbf{Y}}_{\theta } (s)^{{\text{T}}} {\mathbf{q}}_{\theta } (t),\quad v(s,t) = {\mathbf{Y}}_{v} (s)^{{\text{T}}} {\mathbf{q}}_{v} (t) $$

where ${\mathbf{q}}_{w} \in {\mathbb{R}}^{{N_{w} \times 1}} ,{\mathbf{q}}_{\theta } \in {\mathbb{R}}^{{N_{\theta } \times 1}}$ and ${\mathbf{q}}_{v} \in {\mathbb{R}}^{{N_{v} \times 1}}$ are the generalised coordinates corresponding to the ${\mathbf{Y}}_{w} \in {\mathbb{R}}^{{N_{w} \times 1}} ,{\mathbf{Y}}_{\theta } \in {\mathbb{R}}^{{N_{\theta } \times 1}}$ and ${\mathbf{Y}}_{v} \in {\mathbb{R}}^{{N_{v} \times 1}}$ shape functions. In this work, the shifted and iteratively generated set of the Chebyshev functions of the first kind constitute the basis as follows,

7 $$ \begin{aligned} h &= - 2\left( \frac{s}{L} \right) + 1  \\ Y_{1} (s) &= 1,\quad Y_{2} (s) = h,\quad Y_{n} (s) = 2hY_{n - 1} (s) - Y_{n - 2} (s) \\ \end{aligned} $$

These functions are scaled with the positive definite functions in $s \in \left[ {0,L} \right]$ to enforce the kinematic boundary conditions, as described in [38] and implemented in [37]. The resulting scaled functions are,

8 $$ {\mathbf{Y}}_{{w_{n} }} (s) = s^{2} Y_{n} (s),\quad {\mathbf{Y}}_{{\theta_{n} }} (s) = sY_{n} (s),\quad {\mathbf{Y}}_{{v_{n} }} (s) = s^{2} Y_{n} (s) $$

These enforce the cantilever boundary conditions in the translational displacements ($w(0,t) = w^{\prime}(0,t) = 0$ and $v(0,t) = v^{\prime}(0,t) = 0$), and the zero-torsional displacement conditions at the root, $\theta (0,t) = 0$.

In this setting, the assembly of the spatial functions and the corresponding generalised coordinates will be considered in the form $${\mathbf{q}} = \left[ {\begin{array}{*{20}c} {{\mathbf{q}}_{w} } & {{\mathbf{q}}_{\theta } } & {{\mathbf{q}}_{v} } \\ \end{array} } \right]^{{\text{T}}} \in {\mathbb{R}}^{N \times 1}$$ where $N = N_{w} + N_{\theta } + N_{v}$.

### Methods and general structure

The structural model of the overall aeroelastic problem is generated using the Lagrangian equation of the second kind. These can be described compactly in the form,

9 $$ \frac{{\text{d}}}{{{\text{d}}t}}\partial_{{{\dot{\mathbf{q}}}}} \left( {\text{L}} \right)^{{\text{T}}} { = }\partial_{{\mathbf{q}}} \left( {\text{L}} \right)^{{\text{T}}} { + }{\mathbf{Q}}_{{{\text{aero}}}} + {\mathbf{Q}}_{{{\text{grav}}}} $$

where,

10 $$ \partial_{{\mathbf{q}}} \left( {} \right) = \left[ {\begin{array}{*{20}c} {\frac{\partial }{{\partial q_{1} }}} & \cdots & {\frac{\partial }{{\partial q_{N} }}} \\ \end{array} } \right],\quad \partial_{{{\dot{\mathbf{q}}}}} \left( {} \right) = \left[ {\begin{array}{*{20}c} {\frac{\partial }{{\partial \dot{q}_{1} }}} & \cdots & {\frac{\partial }{{\partial \dot{q}_{N} }}} \\ \end{array} } \right] $$

are ${\mathbb{R}}^{R \times 1} \to {\mathbb{R}}^{R \times N}$ operators, L is the Lagrangian assembled as,

11 $$ {\text{L}} = {\text{T}}_{{\text{K}}} - {\text{U}} $$

where ${\text{T}}_{{\text{K}}}$ and ${\text{U}}$ respectively are the total kinetic and the potential energies of the wing described in “Appendix B”, and ${\mathbf{Q}}_{{{\text{aero}}}} ,{\mathbf{Q}}_{{{\text{grav}}}} \in {\mathbb{R}}^{N \times 1}$ are the generalised force vectors corresponding to the aerodynamic and gravitational loading loads.

These structural energy formulations in “Appendix B” are developed using the nonlinear geometrical results that describe the structural motions in Sect. 3.1. Nonlinearity from these expressions arise as higher order polynomial terms. These arise from the power series approximations used to develop the underlying geometrical relationships in “Appendix A”. As noted in “Appendix B”, the nonlinear model used in this implementation intends to retain terms up to and including cubic powers in **q** (in the final equations), and the energy formulations are truncated appropriately to meet this. This choice is based on the moderate deflection levels expected with the examined experimental demonstrator (tip deflections ~ 15% semispan)—previous implementation of the same model in [30] has showed the ability to model similar extents of deflections with the same truncation order. A similar truncation was applied in the model developed in [3]. This was based on a similar approach describing translational and torsional displacements. Here, the model with a truncation applied to retain up to quadratic terms was shown to be capable to model similar levels of deflections [39]. The retaining of terms up to cubic powers in the present model is seen as a computationally feasible extension of the model’s reliability to model the problem at hand.

The generalised forces are obtained by considering the product between the physical force **f** (e.g., vector describing the *x–y–z* components in *O*) and the infinitesimal displacements resulting at the point of action under perturbations of the individual generalised coordinates. That is, if the force acts on a point as described by Eq. (5), the generalised expression can be written as,

12 $$ {\mathbf{Q}} = \partial_{{\mathbf{q}}} \left( {\mathbf{r}} \right)^{{\text{T}}} {\mathbf{f}} $$

This process will result in a system of *N* second order nonlinear differential equations describing the wing’s structural dynamics. The generalised due to gravity is described in “Appendix C”. The next section develops the aerodynamic formulation implemented in this model and is ultimately coupled using the corresponding generalised force.

### 3D unsteady aerodynamics

**Fig. 3:**
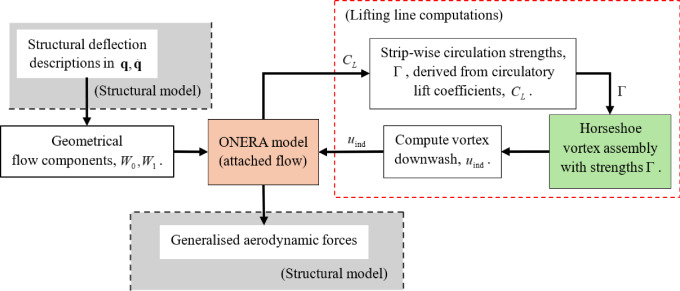
Summary of aerodynamic models and theories integrated in the model

The unique aerodynamic formulation implemented in this modelling framework combines the unsteady circulatory lift modelled by the attached flow component of the ONERA unsteady model [16, 18] (implemented here in the from described in [40]) with the classical Lifting Line Method (LLM) [20, 31] to capture the finite span effects—The implemented theories and models are summarised in Fig. 3. The attached flow equation from the ONERA model is favoured given its feature of being derived with semi-empirically established coefficients. This retains the possibility for the unsteady aerodynamics to be tuned such that the experimentally observed dynamic behaviour (flutter, modal characteristics) is reflected, with an appreciation of possible effects from viscous effects, aerofoil-specific effects, etc. Moreover, the adopted ONERA-based approach retains the possibility of extending the model to capture post-stall dynamics in future implementations.

The primary advantage of this approach, in view of the modelled flow physics, is the ability to capture the finite span effects with unsteady aerodynamics whilst retaining low-order and compact qualities of the model. Particularly, in retrospect of the observations with the Pazy wing model in [19], the underlying flutter-instability mechanisms can be highly sensitive to these effects. As such, the adopted approach is proposed as a novel and generally useful methodology that can capture key physical behaviours at modest computational cost.

This section first describes the spanwise discretisation used to evaluate the aerodynamic loads. Following this, the induced flow components used to compute the aerodynamic loads are defined, using the geometrical results in Sect. 3.1, and “Appendix A”. Finally, the unsteady formulation is described, leading to the development of the generalised loading.

#### Aerodynamic discretisation

**Fig. 4:**
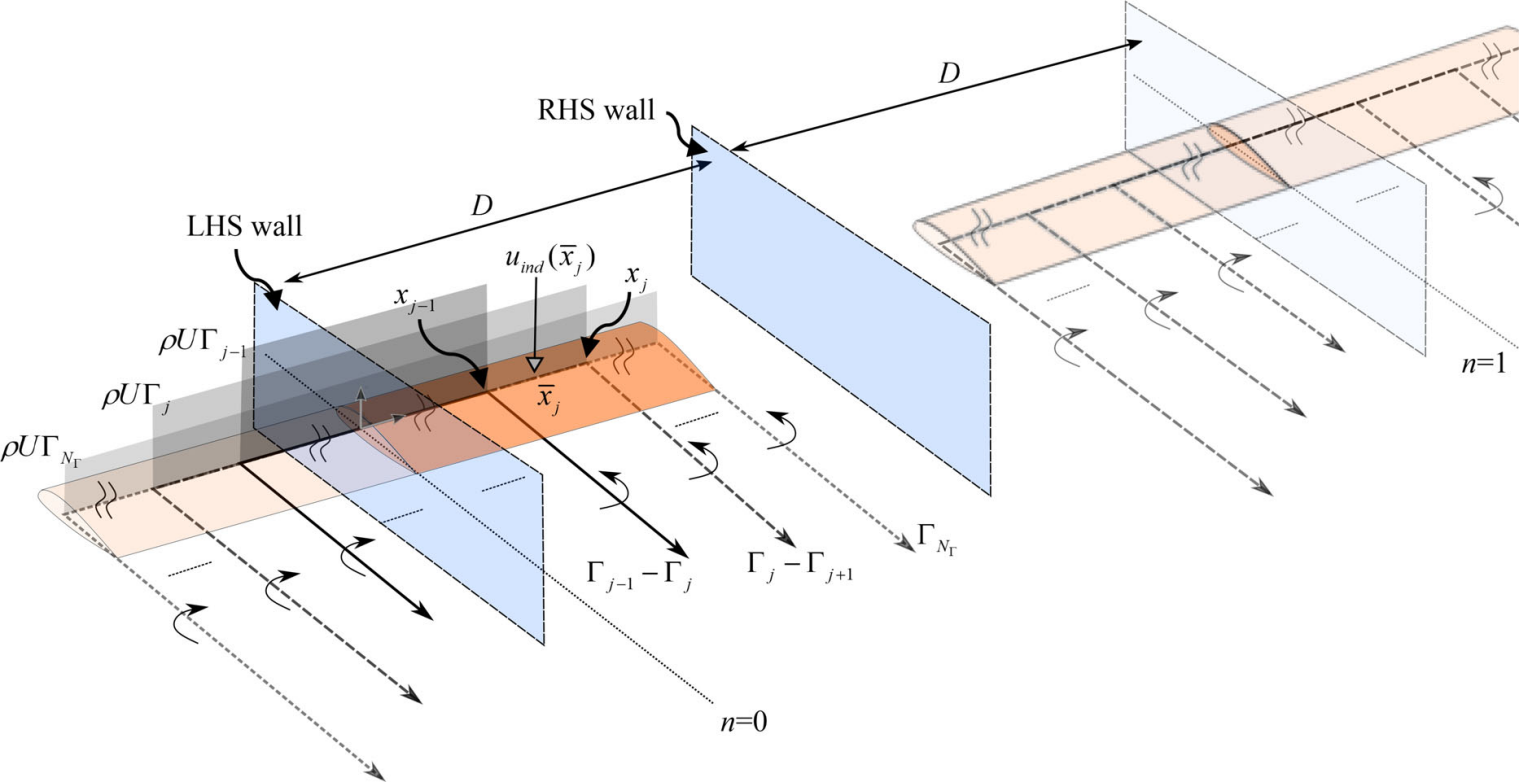
Implementation of the Lifting Line Method (LLM). The physical wing and the two tunnel walls are illustrated with emphasised colours. Only one reflection (*n* = 1) of the horse-shoe vortex blocks is shown

The semi-span of the wing is discretised into $N_{\Gamma }$ strips along its arc length, with the outboard boundary of the *j*-th strip being placed at $s = x_{j}$ as indicated on the ‘physical wing’ in Fig. 4. The strips are equally distributed in $s \in [0,L]$, with $x_{{N_{\Gamma } }} = L$,$x_{0} = 0$ and with each strip being of the length $\Delta x$. The collocation point on each strip used to evaluate the local flow angle is placed at $\overline{x}_{j} = 0.5\left( {x_{j} + x_{j - 1} } \right)$. The computed (strip-wise) local aerodynamic loads are applied on the wing at the same collocation point. The outboard boundary of each strip also marks the position at which the trailing vorticity is released via a discrete horseshoe vortex pertaining to the LLM description. This will be detailed later in this section.

The developments that follow will refer to the induced flow terms and the lift generated at the localised collocation stations. Where geometrical results from Sect. 3.1 and “Appendix A” are carried forward in this process, the geometrical terms will be evaluated at the respective spanwise positions $s = \overline{x}_{j}$.

#### Induced flow definitions and relationships

The uniform upstream flow field is considered to be parallel to the *y–z* plane of *O* and placed at an angle $\alpha_{0}$ from the *y* axis. The wing root angle of attack, excluding the vortex downwash, is also $\alpha_{0}$ and the far field flow is perpendicular to gravity, see Fig. 2. Hence, the far field flow velocity can be written as $${\mathbf{U}}_{\infty } = \left[ {\begin{array}{*{20}c} 0 & { - U\cos \alpha_{0} } & {U\sin \alpha_{0} } \\ \end{array} } \right]^{{\text{T}}}$$, where $U$ is the free stream velocity relative to the fixed frame.

The flow induced at the wing is treated as two separate components termed the *geometrical flow component* (combination of $\alpha_{0}$ and flow induced due to structural motions) and the *vortex downwash* (downwash from the trailing vortices). These inflow terms are evaluated at each collocation point and will be denoted either by a subscript *j* for the *j*-th collocation point, or as a function of $\overline{x}_{j}$ itself. Where geometrical attributes defined in Sect. 3.1 and “Appendix A” are recalled, they are evaluated for the respective collocation points.

The vortex downwash flow, $u_{ind} (\overline{x}_{j} )$, at a local spanwise location is assumed to act in the normal-to-chord direction (along $- {\mathbf{e}}_{{\mathbf{z}}}$). The chordwise variation of this component is neglected in this work. It is assumed that this flow component only varies along the span.

The geometrical flow induced at the EA at a selected spanwise position is given by,

13 $$ {\mathbf{U}}_{{{\text{EA}}_{j} }}^{{{\text{str}}}} = - {\dot{\mathbf{r}}}_{{{\text{EA}}_{j} }} + {\mathbf{U}}_{\infty } $$

In order to establish the local angularity features necessary to derive the aerodynamic loads, the first step is to determine the geometrical flow velocity at the 25% chord, in the normal-to-chord direction (along ${\mathbf{e}}_{{\mathbf{z}}}$), $W_{0j}$. This is given by,

14 $$ W_{{0_{j} }} = {\mathbf{e}}_{{\mathbf{z}}}^{{\text{T}}} {\mathbf{U}}_{{{\text{EA}}_{j} }}^{{{\text{str}}}} - b\left( {a + \frac{1}{2}} \right){{\varvec{\Omega}}}^{{\text{T}}} {\mathbf{e}}_{{\mathbf{x}}} $$

as a superposition of the contribution of the geometrical flow at the EA and the effect of the rotation of the rigid arm between the EA and the quarter chord.

Secondly, the difference of the normal flow induced at the 25% and the 75% positions along the chord, $W_{{1_{j} }}$, is recognised—this quantity is indicative of the rotational velocity of the aerofoil, as seen in [40]. Note that this component is purely of structural origin as the vortex downwash is assumed to be fixed along each chord. This is given by,

15 $$ W_{{1_{j} }} = b{{\varvec{\Omega}}}^{{\text{T}}} {\mathbf{e}}_{{\mathbf{x}}} $$

To correctly establish the aerodynamic loads that act within the cross-sectional planes $\Pi$, the projection of the flow velocity on to this plane is required. This can simply be established by projecting the net flow vector encountered by the EA in the $- {\mathbf{e}}_{{\mathbf{y}}}$ and ${\mathbf{e}}_{{\mathbf{z}}}$ directions that span $\Pi$. These components are given, respectively, by $U_{{\Pi_{j} }}^{ - y}$ and $U_{{\Pi_{j} }}^{z}$ as follows,

16 $$ U_{{\Pi_{j} }}^{ - y} = - {\mathbf{e}}_{{\mathbf{y}}}^{{\text{T}}} {\mathbf{U}}_{{{\text{EA}}_{j} }}^{{{\text{str}}}} ,\quad U_{{\Pi_{j} }}^{z} = {\mathbf{e}}_{{\mathbf{z}}}^{{\text{T}}} {\mathbf{U}}_{{{\text{EA}}_{j} }}^{{{\text{str}}}} - u_{ind} (\overline{x}_{j} ) $$

The magnitude of the flow velocity in $\Pi$ can then be obtained by,

17 $$ U_{{\Pi_{j} }} = \sqrt {\left( {U_{{\Pi_{j} }}^{ - y} } \right)^{2} + \left( {U_{{\Pi_{j} }}^{z} } \right)^{2} } $$

#### Circulatory lift and vortex modelling

Next, the unsteady circulatory lift coefficients, $C_{{L_{j} }} (t)$, at each of the collocation stations and the corresponding horseshoe vortex strengths (illustrated in Fig. 4), $\Gamma_{j} (t)$, are described. These are explicitly defined as a set of $N_{\Gamma }$ states for $${\mathbf{C}}_{{\mathbf{L}}} = \left[ {\begin{array}{*{20}c} {C_{{L_{1} }} } & \cdots & {C_{{L_{{N_{\Gamma } }} }} } \\ \end{array} } \right]^{{\text{T}}}$$, and another set of $N_{\Gamma }$ states for $${{\varvec{\Gamma}}} = \left[ {\begin{array}{*{20}c} {\Gamma_{1} } & \cdots & {\Gamma_{{N_{\Gamma } }} } \\ \end{array} } \right]^{{\text{T}}}$$. These vortex strengths are derived to approximate the downwash distribution, $u_{ind} (\overline{x}_{j} )$, to account for the finite-span effects.

The classical LLM comprises of a single set of horseshoe vortices (just the *n* = 0 block indicated in Fig. 4). This is intended for a free-flying rigid aircraft as the imposed symmetry conditions assume the absence of asymmetrical dynamics between the port and the starboard wings. This is also applicable to a semi-span wing attached to a single wall (‘LHS wall’ at the wing root), as the symmetry of the horseshoes and the ‘pseudo’ reflection of the physical wing about the wall imposes the non-penetration condition of the flow across the wall.

Given the context of the wind tunnel in the present work that comprises of the second (RHS) wall outboard of the wing tip, a modification is required to impose the same non-penetration condition across this wall. This can be realised by implementing an infinite series of pseudo repetitions of the blocks in $\left[ { - \infty ,\infty } \right]$ of $n \in {\mathbb{N}}$, as indicated in Fig. 4. Note that only the instances where *n* = 0 and *n* = 1 is exemplified there. This strategy captures the distribution of the shed vortices that complies with the spatial constraints from the RHS wall, placed at a finite clearance *D-L* from the wing tip (*D* being the width of the tunnel).

Based on the lifting line theory, the strength of a shed vortex is equal to the difference, $\Delta \Gamma_{i}$, of the bound vortices between adjacent strips ($\Delta \Gamma_{{\text{i}}} = \Gamma_{{\text{i}}} - {\Gamma }_{i + 1}$ as illustrated in Fig. 4). The downwash contribution on a collocation point from a single trailing vortex released at a distance $d$ measured along the *O*(*y*) direction from the collocation point equates to $\Delta \Gamma /(4\pi d)$ [20]. Hence, the vortex downwash at the *j*-th collocation point (at $\overline{x}_{j}$) induced by all the trailing vortices can be written as follows,

18 $$ u_{ind} (\overline{x}_{j} ) = \sum\limits_{n = - \infty }^{\infty } {\frac{1}{4\pi }\sum\limits_{i = 1}^{{N_{\Gamma } }} {\left( {{\Gamma }_{i} - {\Gamma }_{i + 1} } \right)\left( {\frac{1}{{x_{i} + 2Dn - \overline{x}_{j} }} + \frac{1}{{x_{i} - 2Dn + \overline{x}_{j} }}} \right)} } ,\quad \Gamma_{{N_{\Gamma } + 1}} = 0 $$

In the above, the outer summation in *n* counts through each block comprising of the symmetric horseshoe ensemble arising from the reflection of the wing about a wall. The physical wing is only present on the RHS for the *n* = 0 block. The inner summation in *i* counts through the pairs of the trailing vortices of each block that are shed at the outboard boundary of the *i*-th strip and its symmetric reflection. The above expression can be compactly written as,

19 $$ {\mathbf{u}}_{{{\mathbf{ind}}}} = {\mathbf{Ad\Gamma }} $$

where $${\mathbf{u}}_{{{\mathbf{ind}}}} = \left[ {\begin{array}{*{20}c} {u_{ind} (\overline{x}_{1} )} & \cdots & {u_{ind} (\overline{x}_{{N_{\Gamma } }} )} \\ \end{array} } \right]^{{\text{T}}}$$. The ${\mathbf{A}} \in {\mathbb{R}}^{{N_{\Gamma } \times N_{\Gamma } }}$, ${\mathbf{d}} \in {\mathbb{R}}^{{N_{\Gamma } \times N_{\Gamma } }}$ matrices are given by,

20 $$ \begin{aligned} {\mathbf{d}} & = \left[ {\begin{array}{*{20}c} 1 & { - 1} & 0 & \cdots & 0 \\ 0 & 1 & { - 1} & \cdots & 0 \\ {} & {} & \vdots & {} & {} \\ 0 & \cdots & 0 & 1 & { - 1} \\ 0 & \cdots & 0 & 0 & 1 \\ \end{array} } \right] \\ \left[ {\mathbf{A}} \right]_{j,i} & = \sum\limits_{n = - \infty }^{\infty } {\frac{1}{4\pi }\left( {\frac{1}{{x_{i} + 2Dn - \overline{x}_{j} }} + \frac{1}{{x_{i} - 2Dn + \overline{x}_{j} }}} \right)} \\ & = \overbrace {{\frac{1}{4\pi }\left( {\frac{1}{{x_{i} - \overline{x}_{j} }} + \frac{1}{{x_{i} + \overline{x}_{j} }}} \right)}}^{n = 0} \\ & \quad + \frac{1}{8\pi D}\left( {\Psi \left( {1 - \frac{{x_{i} - \overline{x}_{j} }}{2D}} \right) - \Psi \left( {1 + \frac{{x_{i} - \overline{x}_{j} }}{2D}} \right) + \Psi \left( {1 - \frac{{x_{i} + \overline{x}_{j} }}{2D}} \right) - \Psi \left( {1 + \frac{{x_{i} + \overline{x}_{j} }}{2D}} \right)} \right) \\ \end{aligned} $$

where $\Psi \left( \bullet \right)$ is the Digamma Function.^1^ This result can be derived by invoking the identity^2^ 6.3.16 from [41]. Note that the implementation in the present model assumes that the shed wake is flat as the deformation of the bound vortex with the wing is not modelled. This assumption is made as only moderate displacements are examined here.

As described in [20] and implemented in [31], the horseshoe vortex strengths, $\Gamma_{j} (t)$, are related to the unsteady circulatory lift coefficients through a first order dynamics captured by the following system of ordinary differential equations (ODE),

21 $$ \frac{2}{{U_{{\Pi_{j} }}^{2} }}\dot{\Gamma }_{j} + \frac{1}{{U_{{\Pi_{j} }} b}}\Gamma_{j} = C_{{L_{j} }} $$

The unsteady circulatory lift coefficients are modelled based on the attached flow components of the ONERA model, given by the ODE system [40],

22 $$ \begin{aligned} \frac{b}{{U_{{\Pi_{j} }} }}\dot{C}_{{L_{j} }} + \lambda_{L} C_{{L_{j} }} & = \lambda_{L} c_{l} \left( {\alpha_{j} ,U_{{\Pi_{j} }} } \right) + \lambda_{L} c_{{l_{\alpha } }} \left( {\alpha_{j} ,U_{{\Pi_{j} }} } \right)W_{{1_{j} }} \\ & \quad + \alpha_{L} \frac{b}{{U_{{\Pi_{j} }} }}c_{{l_{\alpha } }} \left( {\alpha_{j} ,U_{{\Pi_{j} }} } \right)\left( {\dot{W}_{{1_{j} }} + \dot{W}_{{0_{j} }} - \dot{u}_{ind} (\overline{x}_{j} )} \right) \\ \end{aligned} $$

Note that in the above equation, the vortex downwash term is superposed with the normal to chord velocity terms. The terms $\lambda_{L} ,\alpha_{L}$ are semi-empirical coefficients characterising the unsteady behaviour and the tuned values specific to the present numerical investigation will be presented later. $c_{l} \left( {\alpha_{j} ,U_{{\Pi_{j} }} } \right)$ is the steady 2D lift coefficient and $c_{{l_{\alpha } }} \left( {\alpha_{j} ,U_{{\Pi_{j} }} } \right)$ is the gradient of the same quantity with respect to the angle of attack. They are described in “Appendix B” and are taken as functions of the airspeed and the local angle of attack, $\alpha_{j}$, at the 25% chord position of the *j*-th spanwise collocation station. This is approximated using the sine ratio,

23 $$ \alpha_{j} = \frac{{W_{{0_{j} }} - u_{ind} (\overline{x}_{j} )}}{{U_{{\Pi_{j} }} }} $$

Note that the above is evaluated by summing up both the geometrical flow and the vortex downwash components.

#### Aerodynamic forces

The application-specific geometric layout of the aerodynamic forces is developed next. The three net aerodynamic loads applied at each station comprise of lift, drag and the pitching moment. They are referenced to the 25% chord location of each station. The corresponding coefficients are given by [32, 40],

24 $$ \begin{aligned} \overline{C}_{{L_{j} }}^{{}} & = C_{{L_{j} }}^{{}} + \frac{b}{{U_{{\Pi_{j} }} }}\left( {2\pi \dot{W}_{{0_{j} }} + \frac{\pi }{2}\dot{W}_{{1_{j} }} } \right) \\ \overline{C}_{{M_{j} }}^{{}} & = c_{M} \left( {\alpha_{j} ,U_{{\Pi_{j} }} } \right) + \frac{b}{{U_{{\Pi_{j} }}^{2} }}\left( {\overline{\sigma }_{m} \dot{W}_{{0_{j} }} + s_{m} \dot{W}_{{1_{j} }} } \right) + \frac{1}{{U_{{\Pi_{j} }}^{{}} }}\left( {\sigma_{m} W_{{1_{j} }} } \right) \\ \overline{C}_{{D_{j} }}^{{}} & = c_{D} \left( {\alpha_{j} ,U_{{\Pi_{j} }} } \right) \\ \end{aligned} $$

In the above, $\overline{\sigma }_{m}$,$s_{m}$ and $\sigma_{m}$ are the unsteady ONERA coefficients, and $c_{M} \left( {\alpha_{j} ,U_{{\Pi_{j} }} } \right)$ and $c_{D} \left( {\alpha_{j} ,U_{{\Pi_{j} }} } \right)$, respectively, are the static pitching moment and the drag coefficients described in “Appendix B”.

The vector components of the aerodynamic lift, ${\mathbf{f}}_{{{\mathbf{L}}_{j} }} \overline{C}_{{L_{j} }}^{{}}$, and the drag, ${\mathbf{f}}_{{{\mathbf{D}}_{j} }} \overline{C}_{{D_{j} }}^{{}}$ forces are applied, respectively, perpendicular and parallel to the total flow velocity vector projected onto the plane $\Pi$. The ‘aerodynamic force factors’, ${\mathbf{f}}_{{{\mathbf{L}}_{j} }}$ and ${\mathbf{f}}_{{{\mathbf{D}}_{j} }}$, as shown, equates to the aerodynamic force on multiplying with the corresponding force-coefficient. The projected vector terms of the aerodynamic force factors can be written as,

25 $$ {\mathbf{f}}_{{{\mathbf{L}}_{j} }} = b\rho U_{{\Pi_{j} }}^{2} \Delta x\left( {\frac{{U_{{\Pi_{j} }}^{ - y} }}{{U_{{\Pi_{j} }}^{{}} }}{\mathbf{e}}_{{\mathbf{z}}} + \frac{{U_{{\Pi_{j} }}^{z} }}{{U_{{\Pi_{j} }}^{{}} }}{\mathbf{e}}_{{\mathbf{y}}} } \right),\quad {\mathbf{f}}_{{{\mathbf{D}}_{j} }} = b\rho U_{{\Pi_{j} }}^{2} \Delta x\left( { - \frac{{U_{{\Pi_{j} }}^{ - y} }}{{U_{{\Pi_{j} }}^{{}} }}{\mathbf{e}}_{{\mathbf{y}}} + \frac{{U_{{\Pi_{j} }}^{z} }}{{U_{{\Pi_{j} }}^{{}} }}{\mathbf{e}}_{{\mathbf{z}}} } \right) $$

The above expressions, for both lift and drag, essentially projects the corresponding force vector lying in the $\Pi$ plane, on the ${\mathbf{e}}_{{\mathbf{z}}}$ and ${\mathbf{e}}_{{\mathbf{y}}}$ axes that span the same plane. This is done by establishing the cosine ratio of the angle between the force and each of the vectors (${\mathbf{e}}_{{\mathbf{z}}}$,${\mathbf{e}}_{{\mathbf{y}}}$) that are orthogonal to each other: For instance, the cosine of the angles between the lift vector and the ${\mathbf{e}}_{{\mathbf{z}}}$ and ${\mathbf{e}}_{{\mathbf{y}}}$ axes are respectively given by $U_{{\Pi_{j} }}^{ - y} /U_{{\Pi_{j} }}^{{}}$ and $U_{{\Pi_{j} }}^{z} /U_{{\Pi_{j} }}^{{}}$(noting that lift acts perpendicular to projected velocity in $\Pi$, with magnitude $U_{{\Pi_{j} }}^{{}}$). As such, Eq. (25) is geometrically exact and applies the aerodynamic load in a manner that follows the beam deflection (‘follower aerodynamic load’). Nonlinearity here manifests predominantly through the **q**-dependency of the vectors ${\mathbf{e}}_{{\mathbf{z}}}$ and ${\mathbf{e}}_{{\mathbf{y}}}$.

The above loads are applied at the quarter-chord position at each collocation station *j*, such that the position vector of the point of application, ${\mathbf{r}}_{j}^{25\% }$, is

26 $$ {\mathbf{r}}_{j}^{25\% } = {\mathbf{r}}_{{{\text{EA}}}} + b\left( {a + \frac{1}{2}} \right){\mathbf{e}}_{{\mathbf{y}}} $$

The aerodynamic moments are applied at the quarter chord points of each collocation station about the local ${\mathbf{e}}_{{\mathbf{x}}}$ axis. The infinitesimal rotational displacement corresponding to this moment is described using the angles defining the rotation sequence $\phi ,\psi ,\theta$ that fixes the orientation of frame *B* (as described in “Appendix A”, where the first rotation $\phi$ is applied about the negative *O*(*z*) axis, the second angle $\psi$ defining the orientation of ${\mathbf{e}}_{{\mathbf{x}}}$ relative to *O*(*x,y*) plane and the final angle $\theta$ describing the acute angle measured along $\Pi$ between the *O*(*x*,*y*) plane and ${\mathbf{e}}_{{\mathbf{y}}}$). By identifying the axes in *B* along which each of the infinitesimal angular rotations takes place ($\delta \phi$ about $- \sin \psi {\mathbf{e}}_{{\mathbf{x}}} - \cos \psi \sin \theta {\mathbf{e}}_{{\mathbf{y}}} - \cos \psi \cos \theta {\mathbf{e}}_{{\mathbf{z}}}$, $\delta \psi$ about $- \cos \theta {\mathbf{e}}_{{\mathbf{y}}} + \sin \theta {\mathbf{e}}_{{\mathbf{z}}}$, and $\delta \theta$ about ${\mathbf{e}}_{{\mathbf{x}}}$), the net infinitesimal rotation that takes place about the ${\mathbf{e}}_{{\mathbf{x}}}$ axis along which the aerodynamic moment acts can be identified as $- \delta \phi \sin \varphi + \delta \theta \approx w^{\prime}\delta v^{\prime} + \delta \theta = {\mathbf{p}}_{j}^{{\text{T}}} \delta {\mathbf{q}}$, where,

27 $$ {\mathbf{p}}_{j} = - \partial_{{\mathbf{q}}} \left( \phi \right)^{{\text{T}}} \sin \varphi + \partial_{{\mathbf{q}}} \left( \theta \right)^{{\text{T}}} \approx w^{\prime}\partial_{{\mathbf{q}}} \left( {v^{\prime}} \right)^{{\text{T}}} + \partial_{{\mathbf{q}}} \left( \theta \right)^{{\text{T}}} $$

With these forces and the displacements identified, the overall generalised forces due to these loads across all spanwise aerodynamic stations can be written as,

28 $$ \begin{aligned} {\mathbf{Q}}_{{{\text{aero}}}} & = \sum\limits_{j = 1}^{{N_{\Gamma } }} {\partial_{{\mathbf{q}}} \left( {{\mathbf{r}}_{j}^{25\% } } \right)^{{\text{T}}} \left( {{\mathbf{f}}_{{{\mathbf{L}}_{j} }} \overline{C}_{{L_{j} }}^{{}} + {\mathbf{f}}_{{{\mathbf{D}}_{j} }} \overline{C}_{{D_{j} }}^{{}} } \right) + 2b^{2} \rho U_{{\Pi_{j} }}^{2} \Delta x\overline{C}_{{M_{j} }}^{{}} {\mathbf{p}}_{j} } \\ & = \left[ {\begin{array}{*{20}c} \ldots & {\partial_{{\mathbf{q}}} \left( {{\mathbf{r}}_{j}^{25\% } } \right)^{{\text{T}}} {\mathbf{f}}_{{{\mathbf{L}}_{j} }} } & \cdots \\ \end{array} } \right]\left[ {\begin{array}{*{20}c} \vdots \\ {\overline{C}_{{L_{j} }}^{{}} } \\ \vdots \\ \end{array} } \right] + \left[ {\begin{array}{*{20}c} \ldots & {\partial_{{\mathbf{q}}} \left( {{\mathbf{r}}_{j}^{25\% } } \right)^{{\text{T}}} {\mathbf{f}}_{{{\mathbf{D}}_{j} }} } & \cdots \\ \end{array} } \right]\left[ {\begin{array}{*{20}c} \vdots \\ {\overline{C}_{{D_{j} }}^{{}} } \\ \vdots \\ \end{array} } \right]  + \left[ {\begin{array}{*{20}c} \cdots & {2b^{2} \rho U_{{\Pi_{j} }}^{2} \Delta x{\mathbf{p}}_{j} } & \cdots \\ \end{array} } \right]\left[ {\begin{array}{*{20}c} \vdots \\ {\overline{C}_{{M_{j} }}^{{}} } \\ \vdots \\ \end{array} } \right] \\ \end{aligned} $$

The latter expression assembles the generalised aerodynamic forces such that the aggregation of the force coefficients from individual strips are assembled in the $N_{\Gamma } \times 1$ upright vector (the latter part of each term in the expression). The projected vectors of the forces/moment factors on the generalised coordinates are assembled column-wise in the pre-multiplied $N \times N_{\Gamma }$ matrices. This compact format is carried forward to describe the assembly of the complete model in Sect. 3.5.2.

#### ONERA unsteady coefficients

This section presents the unsteady aerodynamic parameters used with the implemented ONERA formulation. These coefficients are strongly dependent on the aerofoil and the flow conditions (in particular, the Reynolds number). A wide range of experimentally determined parameters for applications with several flow conditions and aerofoils have been used extensively for ONERA-based models. In the present implementation, due to the lack of parameters established specifically for the present test conditions and chosen NACA 0018 aerofoil, the values were adjusted such that a good reflection of the experimentally characterised dynamic behaviour is captured. The applied parameter values used in this work are presented in Table 3, along comparison against example values from literature corresponding to several flow conditions and aerofoils to contextualise the appropriateness of the used values.

**Table 3:** ONERA coefficients

Coefficient		Value—present work	Value—used for NACA0012 [6, 32]	Value—based on OA212 data [16, 18]	Value—measured in [43]
Lift (Eq. 22)	$\alpha_{L}$	0.44	0.50	0.44	–
	$\lambda_{L}$	0.275	0.15	0.20	0.25
Moment (Eq. 24)	$\overline{\sigma }_{m}$	$- 0.25\pi$	$- 0.25\pi$	–	–
	$s_{m}$	− 0.589	− 0.589	–	–
	$\sigma_{m}$	$- 0.25\pi$	$- 0.25\pi$	–	–

The first compared example includes one that was used for a wing with a NACA 0012 aerofoil that was derived based on a single-lag approximation to the Theodorsen function (which assumes inviscid flow) [42]. The second example was derived experimentally for the OA212—a noteworthy comparison of the lift response under a step (angle) input with these parameters on the ONERA model against the equivalent with the Wagner’s model (alternative inviscid theory) is offered in [18]. The final example extracted from [43] includes a case with a lower Reynold’s number.

The values used in the present work were tuned with a focus on capturing experimentally characterised aeroelastic modal properties, particularly the damping behaviour—The sensitivity of these dynamic features to the ONERA parameters are discussed in Sect. 6.2, with the context of flow condition dependent unsteady lift behaviours recognised from literature. The tuning was done on the two coefficients affecting the lift coefficient, with the parameters defining the aerodynamic moment adopted from the first example in [6], also used in [32]. As seen on comparing the implemented values against the above examples, the utilised values remain in the vicinity of parameter values typically seen in literature. As such, the tuning-based approach is deemed appropriate for the context of the present demonstration and, as extensively shown in the results section that follows, generates a good replication of experimentally observed modal characteristics and instability features.

### Overview of the model and the state space representation

#### Structural problem and damping

First, the equations of motion corresponding to the aero-off structural problem are introduced. This concerns the energy formulations from “Appendix B”, with the gravitational forces developed in “Appendix C”, applied through the Lagrangian framework described in Sect. 3.3. The resulting system of equations can be structured in the form,

29 $$ {\mathbf{M}}_{{{\text{str}}}} ({\mathbf{q}}){\ddot{\mathbf{q}}} = {\mathbf{F}}_{{{\text{str}}}} ({\mathbf{q}},{\dot{\mathbf{q}}}) $$

where ${\mathbf{M}}_{{{\text{str}}}}$ is the structural mass matrix. The terms in the mass matrix are identified by selecting the expressions resulting from the application of the Lagrangian operator to ${\text{T}}_{{\text{K}}}$ that yields terms that are products of ${\ddot{\mathbf{q}}}$. As discussed in relation to Eq. (62), all terms in ${\text{T}}_{{\text{K}}}$ are quadratic in the generalised velocities, ${\dot{\mathbf{q}}}$. Noting this, the mass matrix can be identified to only depend on **q**. These terms that appear as products with generalised accelerations are separately identified from applying the Lagrangian operator to ${\text{T}}_{{\text{K}}}$. The remainder included in the vector function ${\mathbf{F}}_{{{\text{str}}}} \in {\mathbb{R}}^{N \times 1}$,

30 $$ {\mathbf{M}}_{{{\text{str}}}} ({\mathbf{q}}) = \partial_{{{\dot{\mathbf{q}}}}} \left( {\partial_{{{\dot{\mathbf{q}}}}} \left( {{\text{T}}_{{\text{K}}} } \right)^{{\text{T}}} } \right),\quad {\mathbf{F}}_{{{\text{str}}}} ({\mathbf{q}},{\dot{\mathbf{q}}}) = \left( {\partial_{{\mathbf{q}}} {\text{L}}} \right)^{{\text{T}}} - \left. {\frac{{\text{d}}}{{{\text{d}}t}}\left( {\partial_{{{\dot{\mathbf{q}}}}} \left( {{\text{T}}_{{\text{K}}} } \right)^{{\text{T}}} } \right)} \right|_{{{\ddot{\mathbf{q}}} = {\mathbf{0}}}} + {\mathbf{Q}}_{{{\text{grav}}}} - {\dot{D\mathbf{q}}} $$

As shown above, for the purposes of this study, ${\mathbf{F}}_{{{\text{str}}}}$ includes a structural damping term, $- \mathbf{D}{\dot{\mathbf{q}}}$, of which the $N \times N$ damping matrix **D** will be introduced later.

The system in Eq. can be numerically linearised about a given static equilibrium ${\mathbf{q}}_{0}$ that satisfies the condition ${\mathbf{F}}_{{{\text{str}}}} ({\mathbf{q}}_{0} ,{\mathbf{0}}) = {\mathbf{0}}$. This process yields the linear mass, ${\mathbf{M}}_{0} = {\mathbf{M}}_{{{\text{str}}}} ({\mathbf{q}}_{0} )$, and the stiffness, ${\mathbf{K}}_{0} = - \partial_{{\mathbf{q}}} \left( {{\mathbf{F}}_{{{\text{str}}}} ({\mathbf{q}},{\mathbf{0}})} \right)_{{{\mathbf{q}} = {\mathbf{q}}_{0} }}$, matrices that permit the small vibration analysis about the equilibrium, ${\mathbf{q}} = {\mathbf{q}}_{0} + {\mathbf{q}}_{\delta }$, in the following form;

31 $$ {\mathbf{M}}_{0} {\ddot{\mathbf{q}}}_{\delta } + {\mathbf{D}\dot{\mathbf{q}}}_{\delta } + {\mathbf{K}}_{0} {\mathbf{q}}_{\delta } = {\mathbf{0}} $$

The damping matrix **D** in this work is realised in the form of the Rayleigh model of proportional damping,

32 $$ {\mathbf{D}} = k_{\beta } {\mathbf{M}}_{0} + k_{\alpha } {\mathbf{K}}_{0} $$

where the linear mass and the stiffness matrices above are obtained for the equilibrium when $\alpha_{0} = 0$ and $k_{\beta } = 0.07,k_{\alpha } = 1 \times 10^{ - 4}$. These values are obtained using a manual tuning process such that the model reflects the damping ratios obtained from the GVT done for the same orientation relative to gravity.

#### Aeroelastic problem and model implementation

The complete nonlinear aeroelastic problem comprises of a second order system of size *N* generated by Eq. (9) with the proportional structural damping from Eq. (32). This is combined with a system of $N_{\Gamma }$ first order equations based on Eq. (22), and a set of $N_{\Gamma }$ first order equations based on Eq. (21). The overall aeroelastic system comprises of a total of $2N + 2N_{\Gamma }$ states. The results in this paper are generated with $N_{w} = 6,N_{\theta } = 4$ and $N_{v} = 4$ spatial functions (*N* = 14), and with $N_{\Gamma } = 20$. The choice of the number of spatial functions was determined such that they are sufficient to capture the structural modes of interest (first two out of plane bending and the first torsion and in plane bending modes). This was confirmed using the convergence of selected (frequency domain) transfer functions and modal frequencies across the corresponding range of frequencies (0–25 Hz)—A comparison of the converged frequency responses against the experiment is offered in Sect. 4.2. The choice of $N_{\Gamma }$ was determined based on the ability to obtain sufficiently converged spanwise lift distributions at selected equilibrium conditions, whilst avoiding excessive numbers of states to facilitate the application of the computational methods used in this work.

All computational studies are implemented in the state space with the state vector composed as $${\mathbf{y}} = \left[ {\begin{array}{*{20}c} {{\mathbf{q}}^{{\text{T}}} } & {{\dot{\mathbf{q}}}^{{\text{T}}} } & {{\mathbf{C}}_{{\mathbf{L}}}^{{\text{T}}} } & {{{\varvec{\Gamma}}}^{{\text{T}}} } \\ \end{array} } \right]^{{\text{T}}}$$. The resulting problem is then expressed in the form,

33 $$ {\mathbf{M}}_{\Sigma } ({\mathbf{y}}){\dot{\mathbf{y}}} = {\mathbf{F}}_{\Sigma } ({\mathbf{y}}) $$

where ${\mathbf{M}}_{\Sigma }$ is the $(2N + 2N_{\Gamma } ) \times (2N + 2N_{\Gamma } )$ state space mass matrix and the right-hand side vector ${\mathbf{F}}_{\Sigma } \in {\mathbb{R}}^{{(2N + 2N_{\Gamma } ) \times 1}}$ accumulates all remaining terms originating from the constituent aeroelastic equations. These quantities are essentially a vector–matrix assembly based on the structural system in Eq. (29), coupled with ${\mathbf{Q}}_{{{\text{aero}}}}$ and the aerodynamic models in Eqs. (22) and (21). During this process, it is necessary to discern the terms to be admitted to the state space mass matrix and the terms to be retained in the right-hand side vector. This is particularly the case due to the ${\ddot{\mathbf{q}}}$-dependency of $\dot{W}_{{0_{j} }} ,\dot{W}_{{1_{j} }}$ terms in the aerodynamic equations Eqs. (22) and (24) that follows in to the generalised forces in Eq. (28). This requirement can be addressed by recognising that the time derivative of these down-wash terms can be written invoking the chain rule for differentiation in the form,

34 $$ \dot{W}_{{0_{j} }} = \partial_{{{\dot{\mathbf{q}}}}} W_{{0_{j} }} {\ddot{\mathbf{q}}} + \partial_{{\mathbf{q}}} W_{{0_{j} }} {\dot{\mathbf{q}}},\quad \dot{W}_{{1_{j} }} = \partial_{{{\dot{\mathbf{q}}}}} W_{{1_{j} }} {\ddot{\mathbf{q}}} + \partial_{{\mathbf{q}}} W_{{1_{j} }} {\dot{\mathbf{q}}} $$

From the above form, the mass-like terms appearing as products of the generalised accelerations can be identified and the dependent terms [from Eqs. (22) and (28) in the assembly Eq. (33)] can be appropriately placed in the global mass matrix. The resulting global systems can be assembled in the form of,

35 $$ {\mathbf{M}}_{\Sigma } = \left[ {\begin{array}{*{20}c} {{\mathbf{I}}_{N \times N} } & {} & {} & {} \\ {} & {{\mathbf{M}}_{str} ({\mathbf{q}}) + {\mathbf{M}}_{aero} ({\mathbf{q}})} & {} & {} \\ {} & {{\mathbf{M}}_{CL} ({\mathbf{q}})} & {{\text{diag}}(b/U_{{\Pi_{j} }} )} & {\alpha_{L} {\text{diag}}(c_{{l_{\alpha (j)} }} b/U_{{\Pi_{j} }} ){\mathbf{Ad}}} \\ {} & {} & {} & {{\text{diag}}(2/U_{{\Pi_{j} }}^{2} )} \\ \end{array} } \right],\quad j = 1,\ldots,N_{\Gamma } $$

with the corresponding right-hand side vector being,

36 $$ {\mathbf{F}}_{\Sigma } = \left[ {\begin{array}{*{20}c} {{\dot{\mathbf{q}}}} \\ {{\mathbf{F}}_{str} + {\mathbf{F}}_{aero} } \\ {\left[ {\begin{array}{*{20}c} \vdots \\ { - \lambda C_{{L_{j} }} + \lambda_{L} c_{l(j)} + \lambda_{L} c_{{l_{\alpha (j)} }} W_{{1_{j} }} + \alpha_{L} bc_{{l_{\alpha (j)} }} \left( {\partial_{{\mathbf{q}}} W_{{0_{j} }} {\dot{\mathbf{q}}} + \partial_{{\mathbf{q}}} W_{{1_{j} }} {\dot{\mathbf{q}}}} \right)/U_{{\Pi_{j} }} } \\ \vdots \\ \end{array} } \right]} \\ {\left[ {\begin{array}{*{20}c} \vdots \\ { - \Gamma_{j} /(U_{{\Pi_{j} }} b) + C_{{L_{j} }} } \\ \vdots \\ \end{array} } \right]} \\ \end{array} } \right],\quad j = 1,\ldots,N_{\Gamma } $$

In the above, ${\mathbf{M}}_{aero} ({\mathbf{q}})$ and ${\mathbf{M}}_{CL} ({\mathbf{q}})$ in the mass-matrix assembly are the terms resulting from the previously discussed generalised acceleration-dependency of the $\dot{W}_{{0_{j} }} ,\dot{W}_{{1_{j} }}$ terms. These, along with the other constituent terms in the above are,

37 $$ \begin{aligned} {\mathbf{M}}_{aero} ({\mathbf{y}}) & = - \left[ {\begin{array}{*{20}c} \ldots & {\partial_{{\mathbf{q}}} \left( {{\mathbf{r}}_{j}^{25\% } } \right)^{{\text{T}}} {\mathbf{f}}_{{{\mathbf{L}}_{j} }} } & \cdots \\ \end{array} } \right]\left[ {\begin{array}{*{20}c} \vdots \\ {(b/U_{{\Pi_{j} }} )\left( {2\pi \partial_{{{\dot{\mathbf{q}}}}} W_{{0_{j} }} + (\pi /2)\partial_{{{\dot{\mathbf{q}}}}} W_{{1_{j} }} } \right)} \\ \vdots \\ \end{array} } \right] + \\ & \quad - \left[ {\begin{array}{*{20}c} \cdots & {2b^{2} \rho U_{{\Pi_{j} }}^{2} \Delta x{\mathbf{p}}_{j} } & \cdots \\ \end{array} } \right]\left[ {\begin{array}{*{20}c} \vdots \\ {(b/U_{{\Pi_{j} }}^{2} )\left( {\overline{\sigma }_{m} \partial_{{{\dot{\mathbf{q}}}}} W_{{0_{j} }} + s_{m} \partial_{{{\dot{\mathbf{q}}}}} W_{{1_{j} }} } \right)} \\ \vdots \\ \end{array} } \right] \in {\mathbb{R}}^{N \times N} \\ {\mathbf{M}}_{CL} ({\mathbf{y}}) & = \left[ {\begin{array}{*{20}c} \vdots \\ { - \alpha_{L} (b/U_{{\Pi_{j} }} )c_{{l_{\alpha (j)} }} \left( {\partial_{{{\dot{\mathbf{q}}}}} W_{{0_{j} }} + \partial_{{{\dot{\mathbf{q}}}}} W_{{1_{j} }} } \right)} \\ \vdots \\ \end{array} } \right] \in {\mathbb{R}}^{{N_{\Gamma } \times N}} \\ \end{aligned} $$

for $$j = 1,\ldots,N_{\Gamma }$$ and,

38 $$ \begin{aligned} {\mathbf{F}}_{aero} & = \left[ {\begin{array}{*{20}c} \ldots & {\partial_{{\mathbf{q}}} \left( {{\mathbf{r}}_{j}^{25\% } } \right)^{{\text{T}}} {\mathbf{f}}_{{{\mathbf{L}}_{j} }} } & \cdots \\ \end{array} } \right]\left[ {\begin{array}{*{20}c} \vdots \\ {C_{{L_{j} }}^{{}} + \left( {b/U_{{\Pi_{j} }} } \right)\left( {2\pi \partial_{{\mathbf{q}}} W_{{0_{j} }} {\dot{\mathbf{q}}} + (\pi /2)\partial_{{\mathbf{q}}} W_{{1_{j} }} {\dot{\mathbf{q}}}} \right)} \\ \vdots \\ \end{array} } \right] \\ & \quad + \left[ {\begin{array}{*{20}c} \ldots & {\partial_{{\mathbf{q}}} \left( {{\mathbf{r}}_{j}^{25\% } } \right)^{{\text{T}}} {\mathbf{f}}_{{{\mathbf{D}}_{j} }} } & \cdots \\ \end{array} } \right]\left[ {\begin{array}{*{20}c} \vdots \\ {\overline{C}_{{D_{j} }}^{{}} } \\ \vdots \\ \end{array} } \right] \\ & \quad + \left[ {\begin{array}{*{20}c} \cdots & {2b^{2} \rho U_{{\Pi_{j} }}^{2} \Delta x{\mathbf{p}}_{j} } & \cdots \\ \end{array} } \right]\left[ {\begin{array}{*{20}c} \vdots \\ {c_{M(j)} + \left( {b/U_{{\Pi_{j} }}^{2} } \right)\left( {\overline{\sigma }_{m} \partial_{{\mathbf{q}}} W_{{0_{j} }} {\dot{\mathbf{q}}} + s_{m} \partial_{{\mathbf{q}}} W_{{1_{j} }} {\dot{\mathbf{q}}}} \right) + \frac{1}{{U_{{\Pi_{j} }}^{{}} }}\left( {\sigma_{m} W_{{1_{j} }} } \right)} \\ \vdots \\ \end{array} } \right] \in {\mathbb{R}}^{N \times 1} \\ \end{aligned} $$

for $j = 1,\ldots,N_{\Gamma }$. The latter are the ${\ddot{\mathbf{q}}}$-independent terms of the generalised aerodynamic forces from Eq. (28).

The individual blocks in the above equations were developed as MATLAB functions^3^ in this work, along with several dependent terms (such as the flow components from Sect. 3.4.2) that are accessed by the dependent functions. The functions returning individual block components are then called in the assembly functions that return the global forcing vector/matrix. Block component functions which involve expressions that are not explicitly expanded and laid out, and which require additional algebraic manipulations—in particular terms that includes the $\partial_{{\mathbf{q}}}$,$\partial_{{{\dot{\mathbf{q}}}}}$ operators, were developed with the symbolic toolbox in MATLAB^4^ and then converted to MATLAB functions. To enhance the computational performance, the functions returning the ultimate global assemblies were re-generated in C++ and accessed as MATLAB executable^5^ (‘mex’) functions.

## Measurement definitions, reference characteristics and set-up for further analysis

### Measurement definitions

In order to enable the direct comparison of the aeroelastic bifurcation behaviour exhibited by the model and that observed in the experiment, the experimentally derived wing root load responses are calculated by the model. They are selected as suitable candidates for this task as the provide information about both the static aeroelastic responses and the LCO responses. As described in Sect. 2.1, the wing root load measurements comprised of the bending moment, *M*_*y*_, the torsional moment, *M*_*x*_, and the transversal shear load, *S*_*z*_. These were acquired using a series of strain gauges stationed at arc-length wise positions $s = x_{1} = 25\,{\text{mm}}$ and $s = x_{2} = 50\,{\text{mm}}$. In the model, these loads are extracted using the following analytical expressions originating in the classical (Euler–Bernoulli based) engineering theories of beam bending and torsion [44].

39 $$ M_{y} = {\mathbf{V}}_{My} {\mathbf{q}},\quad M_{x} = {\mathbf{V}}_{Mx} {\mathbf{q}},\quad S_{z} = {\mathbf{V}}_{Sz} {\mathbf{q}}, $$

where,

40 $$ {\mathbf{V}}_{My} = EI_{yy} \partial_{{\mathbf{q}}} w^{\prime\prime}(x_{1} ),\quad {\mathbf{V}}_{Mx} = GJ\partial_{{\mathbf{q}}} \theta^{\prime}(x_{2} ),\quad {\mathbf{V}}_{Sz} \approx \frac{{EI_{yy} \partial_{{\mathbf{q}}} w^{\prime\prime}(x_{2} ) - EI_{yy} \partial_{{\mathbf{q}}} w^{\prime\prime}(x_{1} )}}{{x_{2} - x_{1} }} $$

Note that as implemented in the experiment, the shear force is obtained as a finite difference-approximation from the bending moments at the two measurement stations.

### Correlation with the ground test results

The results from the GVT campaign carried out under the wind tunnel conditions in [33] are used here. The tests compared here were completed for a single-input and multiple-output scenario. The modal hammer was used to provide impulsive loading at a position at 35% of the chord from the leading edge on segment #6 [from Fig. 1(a)] in the transversal direction. This impact station coincided with a contact point between the outer fairing and the internal T-interfaces in the wing, eliminating thus the superfluous effects arising from the load transmissions through the PLA fairing. Table 4 compares the experimentally derived modal frequencies from [33] with the model obtained for the wing root pitch setting $\alpha_{0} = 0$ with the wing deformed under its self-weight.

**Table 4:** Modal frequencies and damping ratios of the first four modes at $\alpha_{0} = 0$

Mode	Frequency, [Hz]		Damping ratio, [–]	
	Experiment	Model	Experiment	Model
OOP1	1.9	1.9	0.003	0.003
IP1	8.7	8.8	0.017	0.003
OOP2	11.4	11.5	0.004	0.003
TOR1	16.7	16.9	0.001	0.004

The Rayleigh damping parameters for the model, described in Sect. 3.5.1, was informed by the above experimental values. As only the damping ratios of two modes can be exactly matched with this approach [with the two parameters $k_{\alpha } ,k_{\beta }$, using their relationship with the resulting damping ratio, $\varsigma_{i} = k_{\beta } /(2\omega_{i} ) + k_{\alpha } \omega_{i} /2$ for two modes $i = 1,2$, where $\omega_{i}$ are the modal frequencies [45]], this was done with the experimental values of the OOP1 and OOP2 modes. These modes were selected as this combination resulted in representative damping ratios for all the modes (except for IP1—discussed later) in the frequency range of interest.

A good match is obtained in the frequencies of the first four modes that comprise of the first two out of plane bending modes (labelled OOP1 and OOP2), the first in plane bending mode (IP1) and the first torsional mode (TOR1). The OOP1-2 and the TOR1 modes exhibit relatively low levels of damping (< 1%) and comparable levels of damping are reflected by the model. In the experimental case, owing to its design and boundary constraint specifics, the IP1 mode was noted to be more damped at ~ 1.7% and this is not reflected by the model that features a lower level of damping. This, however, is not expected to affect the aeroelastic instability observed as this was shown to be influenced by the OOP2 and the TOR1 modes [33]. Based on a comparison made with a GVT performed under the controlled laboratory conditions where the wing was mounted more rigidly, the elevated damping associated with the in-plane bending dynamics is assumed to originate from the wind tunnel wing root mounting structure.

**Fig. 5:**
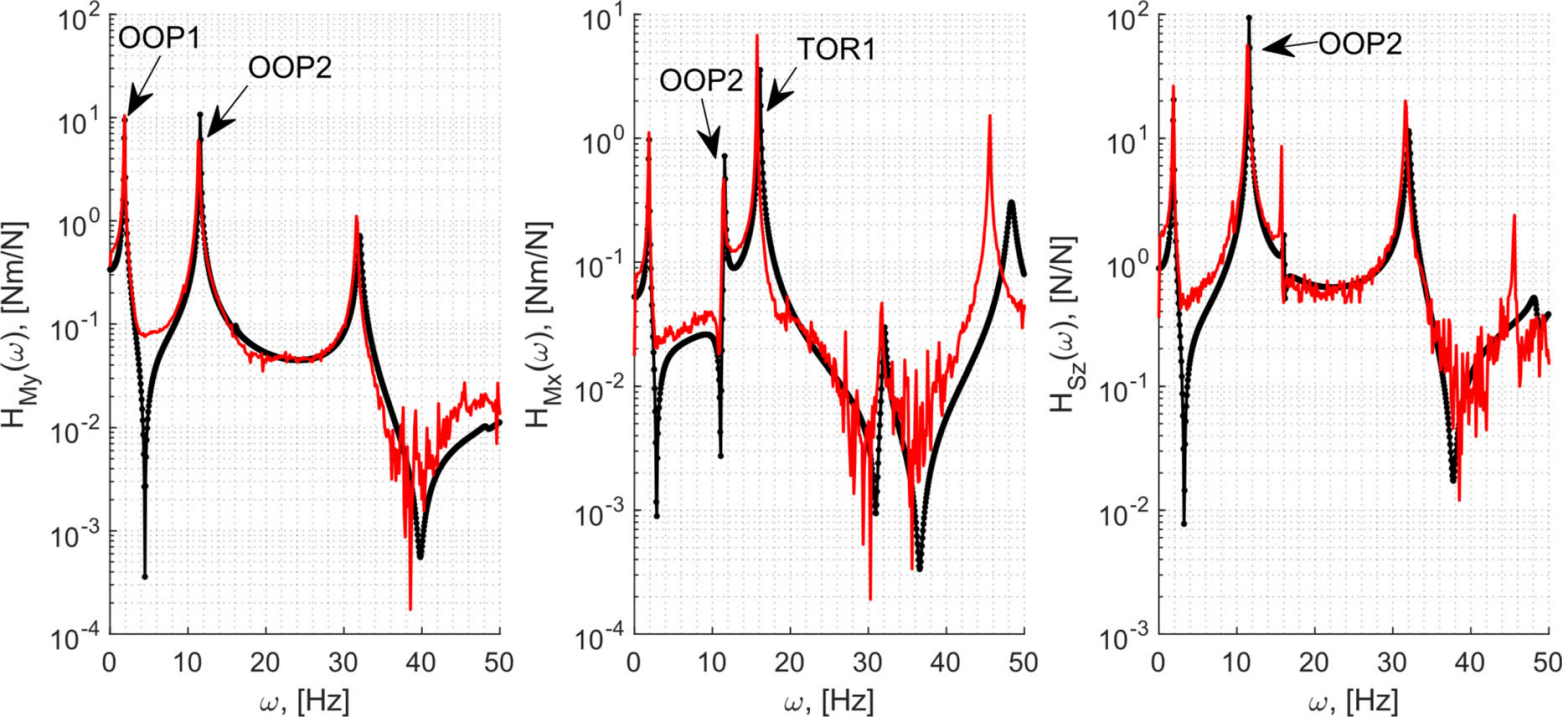
Load transmissibility functions about the $\alpha_{0} = 90^{ \circ }$ equilibrium: red-experiment, black-model

**Fig. 6:**
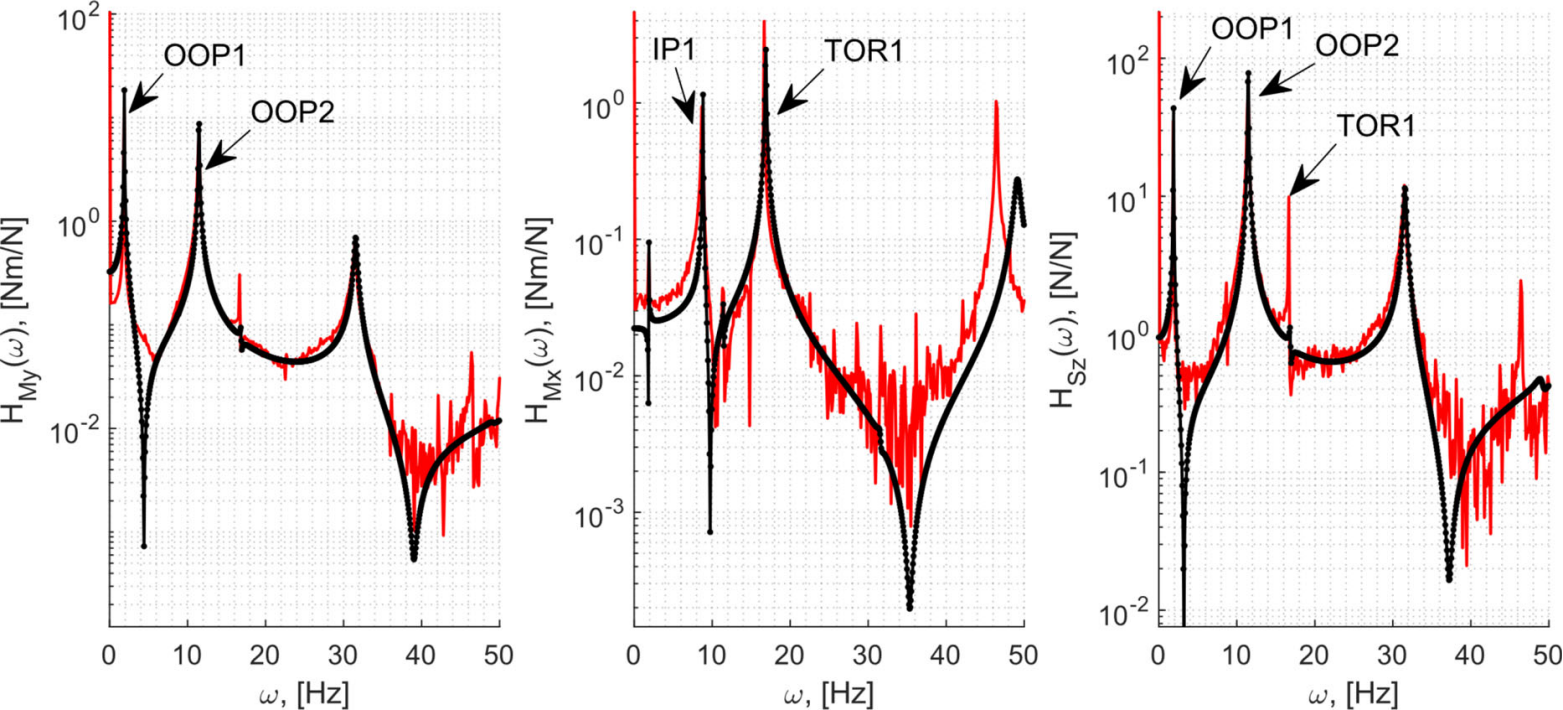
Load transmissibility functions about the $\alpha_{0} = 0$ equilibrium: red-experiment, black-model

To compare the responses exhibited by the wing root load measurements defined previously, Figs. 5 and 6 illustrate the corresponding load transmissibility functions, respectively collected at $\alpha_{0} = 90^{ \circ }$ and $\alpha_{0} = 0$*.* The two static cases reflect conditions where the wing is minimally deflected and a case where a more substantial deflection due to self-weight is present.

As shown in the figures, the model yields a good match with the experimentally determined transfer functions, particularly in the frequency range of the first four modes. A noteworthy and correctly captured feature in these transmissibility functions is the substantial difference of the case of the wing root torsional moment, *M*_*x*_, response across the two static configurations. In particular, the IP1 mode becomes increasingly observable under the deflected scenario ($\alpha_{0} = 0$), whilst the visibility of the OOP2 mode is attenuated.

### Preparation for further analysis

The numerical aeroelastic analysis presented in this paper stems from the application of the numerical continuation routines from ‘coco’ (continuation core) tool [46] implemented in MATLAB. This includes a combination of the equilibria computations, Hopf Bifurcation detection and tracing of the limit cycle response trajectories.

It was generally observed that the problem described in the form shown in Eq. required a significant computational time during the LCO tracing. It was noted that this could be substantially improved by performing a coordinate transformation, to express the original structural degrees of freedom with the mass-normalised modal coordinates. Note that the formulation is still utilised in its nonlinear form, with the structural degrees of freedom described in modal coordinates in lieu of generalised coordinates. The underlying process can be described as follows.

Initially, the nonlinear structural equilibrium problem [Eq. (29)] is solved for the case $\alpha_{0} = 0$, identifying the static solution ${\mathbf{q}}_{0}$. Following this, the undamped eigenvalue problem about the linearised model is solved [Eq. (31), excluding the damping matrix** D**] to establish the *N*-by-*N* $${\mathbf{\varphi }} = \left[ {\begin{array}{*{20}c} {{\mathbf{\varphi }}_{1} } & \cdots & {{\mathbf{\varphi }}_{N} } \\ \end{array} } \right] \in {\mathbb{R}}^{N \times N}$$ ${\mathbf{\varphi }}_{i}^{{\text{T}}} {\mathbf{M}}_{0} {\mathbf{\varphi }}_{i} = 1$. The vectors are arranged in the order of the increasing natural frequencies. The matrix ${\mathbf{\varphi }}$ is then used as a *reference basis* to represent the structural degrees of freedom in the form,

41 $$ {\mathbf{q}}(t) = {\mathbf{q}}_{0} + {\mathbf{\varphi \gamma }}(t) $$

where ${{\varvec{\upgamma}}}(t) \in {\mathbb{R}}^{N \times 1}$ are the new modal coordinates. The first four of these coordinates corresponds to the first four structural modes about the considered equilibrium condition from Table 4 (i.e., ${{\varvec{\upgamma}}}_{1} = {{\varvec{\upgamma}}}_{{{\text{OOP1}}}} ,{{\varvec{\upgamma}}}_{2} = {{\varvec{\upgamma}}}_{{{\text{IP1}}}}$ and so on). To account for the replaced structural coordinates, a new state vector is defined in the form $${\overline{\mathbf{y}}} = \left[ {\begin{array}{*{20}c} {{{\varvec{\upgamma}}}^{{\text{T}}} } & {{\dot{\mathbf{\gamma }}}^{{\text{T}}} } & {{\mathbf{C}}_{{\mathbf{L}}}^{{\text{T}}} } & {{{\varvec{\Gamma}}}^{{\text{T}}} } \\ \end{array} } \right]^{{\text{T}}}$$. Additionally, the structural equations in Eq. (33) (from the *N* + 1-th row, up to the 2*N*-th row) are pre-multiplied by ${\mathbf{\varphi }}^{{\text{T}}}$. As such, the underlying process is only a coordinate transformation and a pre-multiplication of the system of nonlinear equations. The resulting state space mass matrix and the right-hand side vector can be written as,

42 $$ {\overline{\mathbf{M}}}_{\Sigma } = \left[ {\begin{array}{*{20}c} {{\mathbf{I}}_{N \times N} } & {} & {} & {} \\ {} & {{\mathbf{\varphi }}^{{\text{T}}} \left( {{\mathbf{M}}_{str} ({\mathbf{q}}) + {\mathbf{M}}_{aero} ({\mathbf{q}})} \right){\mathbf{\varphi }}} & {} & {} \\ {} & {{\mathbf{M}}_{CL} ({\mathbf{q}}){\mathbf{\varphi }}^{{\text{T}}} } & {{\text{diag}}(b/U_{{\Pi_{j} }} )} & {\alpha_{L} {\text{diag}}(c_{{l_{\alpha (j)} }} b/U_{{\Pi_{j} }} ){\mathbf{Ad}}} \\ {} & {} & {} & {{\text{diag}}(2/U_{{\Pi_{j} }}^{2} )} \\ \end{array} } \right],\quad j = 1,\ldots,N_{\Gamma } $$

43 $$ {\overline{\mathbf{F}}}_{\Sigma } = \left[ {\begin{array}{*{20}c} {{\dot{\mathbf{\gamma }}}} \\ {{\mathbf{\varphi }}^{{\text{T}}} \left( {{\mathbf{F}}_{str} ({\mathbf{q}},{\dot{\mathbf{q}}}) + {\mathbf{F}}_{aero} ({\mathbf{y}})} \right)} \\ {\left[ {\begin{array}{*{20}c} \vdots \\ { - \lambda C_{{L_{j} }} + \lambda_{L} c_{l(j)} + \lambda_{L} c_{{l_{\alpha (j)} }} W_{{1_{j} }} + \alpha_{L} bc_{{l_{\alpha (j)} }} \left( {\partial_{{\mathbf{q}}} W_{{0_{j} }} {\dot{\mathbf{q}}} + \partial_{{\mathbf{q}}} W_{{1_{j} }} {\dot{\mathbf{q}}}} \right)/U_{{\Pi_{j} }} } \\ \vdots \\ \end{array} } \right]} \\ {\left[ {\begin{array}{*{20}c} \vdots \\ { - \Gamma_{j} /(U_{{\Pi_{j} }} b) + C_{{L_{j} }} } \\ \vdots \\ \end{array} } \right]} \\ \end{array} } \right],\quad j = 1,\ldots,N_{\Gamma } $$

Given the orthogonality property ${\mathbf{\varphi }}^{{\text{T}}} {\mathbf{M}}_{str} ({\mathbf{q}}_{0} ){\mathbf{\varphi }} = {\mathbf{I}}_{N \times N}$, this modified system, that continues to describe the original aeroelastic problem, benefits from a generally better-scaled structural mass matrix that leads to more computationally efficient realisation of the continuation analyses. This process is simply an eigenvector-based projection and does not introduce any additional assumptions regarding the physics of the problem or its nonlinear characteristics.

## Aeroelastic bifurcation analysis

**Fig. 7:**
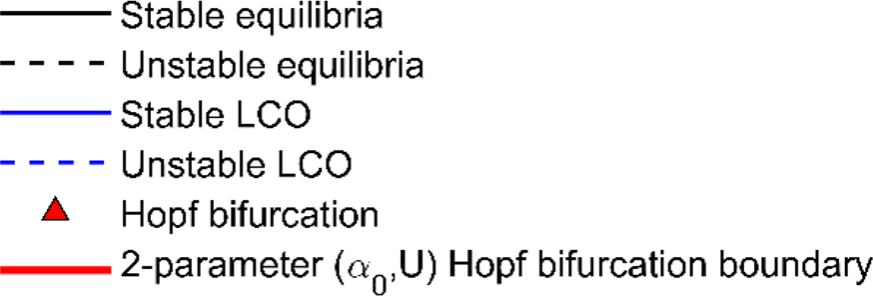
Key for numerical (model-based) bifurcation results

This section presents the numerically generated bifurcation results, and these are compared against those extracted experimentally during the wind tunnel testing. Initially, the static equilibria are introduced, along with the bifurcation boundary defining the emergence of the LCO responses. Following this, the LCO responses are explored, comparing the wing root load responses and frequency/period characteristics against the experiment. To effectively illustrate the numerical results, the key described in Fig. 7 will be used to express all numerical bifurcation results in this section.

### Static aeroelastic responses and stability

**Fig. 8:**
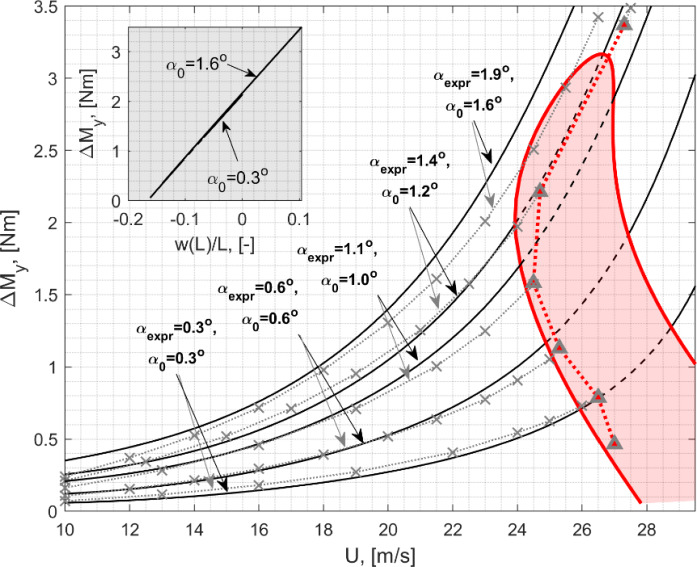
Static aeroelastic responses showing the relationship between $\Delta M_{y}$ and $U$. Numerical results follow the definitions in Fig. 7. The crosses indicate stable equilibria from the experiment, with the Hopf points indicated by the triangle markers. The figure insert shows the relationship between tip deflection levels and $\Delta M_{y}$

To present the static aeroelastic responses under a range of wing root pitch and airspeed combinations, Fig. 8 shows the static wing root bending moment responses. This is done to identify the regions of unstable equilibria mapped by airspeeds and the deflection states indicated by the bending moments. The static load responses are used to facilitate comparison against the experimental results, as this was measured directly during the experiment. These loads are expressed as incremental quantities about that obtained at the wind off (*U* = 0) equilibrium:

44 $$ \Delta M_{y} (U,\alpha_{0} ) = M_{y} (U,\alpha_{0} ) - M_{y} (0,\alpha_{0} ) $$

Furthermore, to provide an indication of the deflection levels, the figure insert shows the model-based relationship between the incremental wing root bending moment at the normalised static tip deflection. This is shown for two selected pitch settings—as suggested by the figure, the deflection vs $\Delta M_{y}$ relationship is approximately invariant of the pitch setting. Note that the tip deflections were not explicitly measured during experimental testing and hence the model-based equivalent is provided as an indicator of this. The wing deflected between just under—15% semi-span under its self-weight and up to just over + 10% semi-span with aerodynamic loading during testing.

The information in the figure, that overlays the computed and the experimental static responses, is arranged such that each curve corresponds to a sweep of *U* with a fixed wing root pitch angle. The wing root pitch angles in the experimental and the numerical counterparts are respectively indicated by $\alpha_{{{\text{expr}}}}$ and $\alpha_{0}$. Note that the model assumes a uniform far field flow that is parallel to the horizontal. In contrast to this, the flow in the working section of the wind tunnel may not be uniform (e.g., varying flow angularities across the working section) and placed at a general angular offset from the horizontal. During testing, the wing root pitch settings in [33] were measured against the horizontal using an inclinometer.

By comparison against model-based results at small pitch angles ($\alpha_{0} = 0.3^{ \circ } ,0.6^{ \circ }$ in the figure), an approximate 0.8-degree difference is gauged between the tunnel’s flow angularity and the pitch angles originally measured against the horizontal. As such, this constant offset is applied when referring to the experimental pitch angles from [33] (e.g. $\alpha_{{{\text{expr}}}} = 1.4^{ \circ }$ refers to an originally measured angle of $2.2^{ \circ }$). This is done to establish an estimate for the general offset of the tunnel’s flow angularity from the horizontal, to facilitate further comparisons.

Each of the $U$ vs $M_{y}$ curves in the figure is presented as a pair of the numerical and experimental counterparts. The corresponding numerical counterparts are computed with values of $\alpha_{0}$ that yield a closely matching static response with changing $U$. Here, it is noted that the predicted static deflection responses are expected to be highly sensitive to the spanwise lift distributions, that is idealised using a lifting line representation. As a result of this approximation in the model and other experimental uncertainties (e.g., related to non-uniform flow), $U$ vs $M_{y}$ responses would not match with same values of $\alpha_{{{\text{expr}}}}$ and $\alpha_{0}$. With the focus on developing a comparison of the regions of unstable equilibria as a function of airspeed and the deflection levels inferred by $\Delta M_{y}$, the pairing of $U$ vs $M_{y}$ curves in the figure was done with values of $\alpha_{{{\text{expr}}}}$ and $\alpha_{0}$ that yielded comparably similar static responses. The resulting discrepancy between $\alpha_{{{\text{expr}}}}$ and $\alpha_{0}$ can be seen to be more prominent at higher deflection levels. This could be indicative of a predictive error linked to the lifting line idealisation where the rate of change of the total aerodynamic load on the wing with the pitch setting (3D lift gradient) is not correctly reflected. The extent of error resulting from this reasoning alone cannot be established conclusively due to the other possible sources of error and experimental uncertainties related to the flow. For instance, given the small pitch angles compared (< 2 degrees), the effect of non-uniform flow angularity in the working section could have a considerable influence. However, the model predicts comparable ranges of static wing root loads across the tested range of airspeeds and adjusted pitch settings to enable meaningful comparisons.

A key focus among the capabilities of the developed model is its ability to reflect the aeroelastic instability thresholds. With the activation of structural geometrical nonlinearity, the instability thresholds (flutter speeds) are known to exhibit rich behaviour with wing deflection dependencies (appearing as pitch angle dependent flutter speeds) as observed in [6, 24]. To identify these behaviours in the context of the present demonstrator, the 2-parameter continuation of the Hopf Bifurcation boundary that enclose the unstable equilibria is indicated in the figure (visualised there on the *U*-*M*_*y*_ domain of equilibria). As seen in the figure, the model yields a good agreement against the experimentally derived instability thresholds. In particular, the computed flutter onset speeds appear within 5% of the experimental values at a given $\Delta M_{y}$ level.

The feature of the airspeed-bounded instability of the equilibrium curves with fixed pitch settings, as they intersect the Hopf Boundary at two points, is indicative of a hump-mode instability mechanism (also observed with the Pazy wing demonstrator [24]). The first intercept of the Hopf boundary marks the flutter speed, at which the LCO responses emerge. As indicated by the shape of the Hopf boundary and the enclosed unstable equilibria, the flutter speed is seen to evolve with varying deflection levels (brought by varying the pitch setting). More specifically, the flutter speed initially drops with increased wing root pitch settings, and then increases gradually as the instability region encloses at higher pitch settings (being flutter-free at pitch settings beyond this). Ultimately, with increasing pitch angles, the instability mechanism ceases to exist. This behaviour suggested by the model is in agreement, both qualitatively and quantitatively, with the experimentally established Hopf bifurcations overlaid in the figure.

### Limit cycle oscillations (LCOs)

With the context of the static aeroelastic responses and the Hopf Bifurcations examined in the previous section, this section extends the comparison to the associated LCO responses. Besides examining the model’s capability to replicate the experimentally established LCO characteristics, this section investigates the role of the implemented aerodynamics formulation in capturing the LCO responses.

**Fig. 9:**
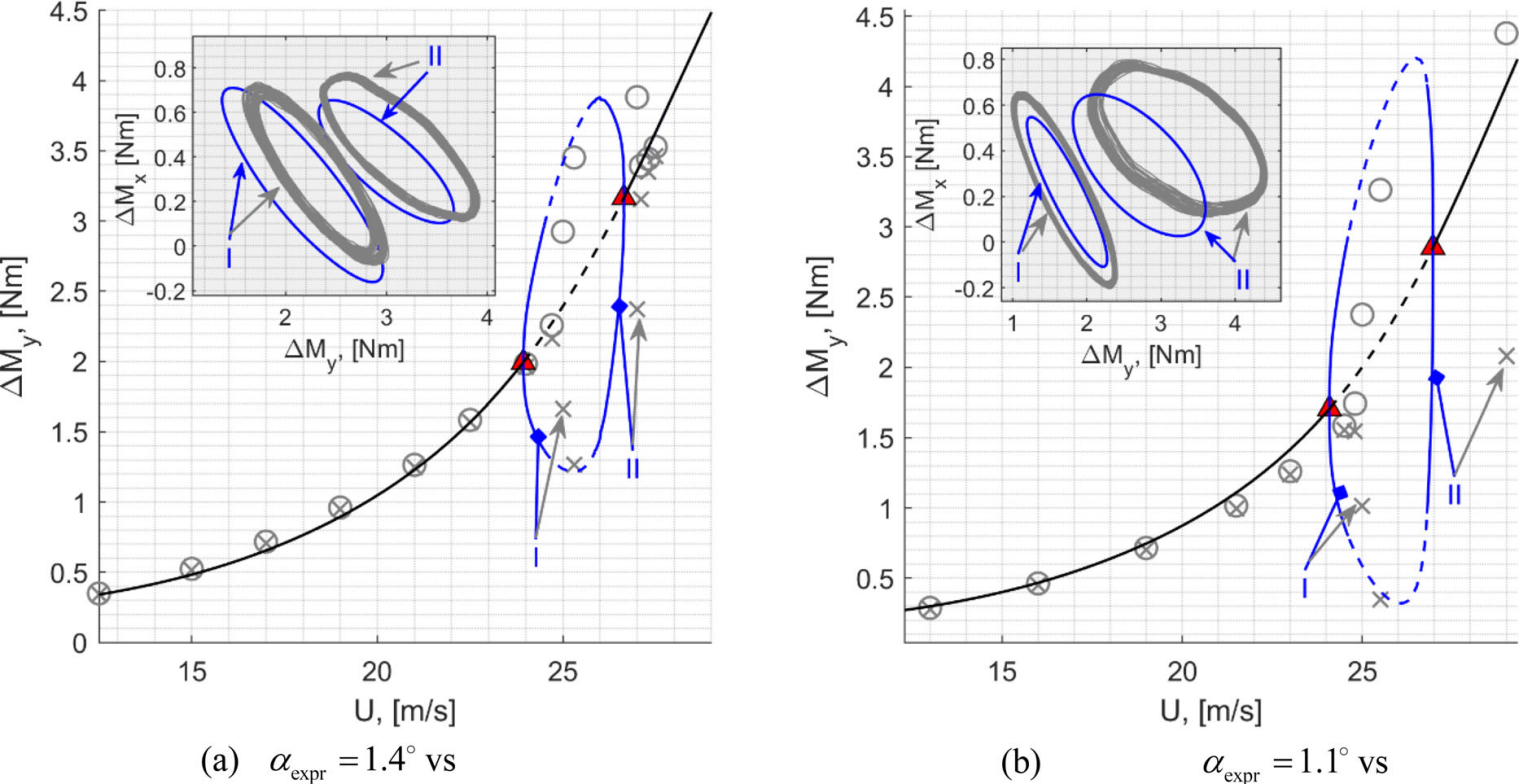
Single parameter (airspeed) bifurcation diagrams comparing the numerical results (following the key in Fig. 7) against the experiment indicated by the circle and cross markers. The selected LCO orbits in *M*_*x*_*–M*_*y*_ are exemplified in the figure inserts

The complete 1-parameter airspeed bifurcation behaviour that includes both the equilibrium and LCOs is shown in Fig. 9 for two selected wing root pitch settings. The experimental results across the stable equilibria and LCO responses are indicated using the maxima (circles) and the minima (crosses) of $M_{y}$ from the undisturbed acquisitions. At stable equilibria, the markers for maxima and minima merge, indicating the absence of self-excited motion. In the LCO regions, the vertical separation of the markers indicates peak-to-peak amplitude of the $M_{y}$ response in the LCO. The numerical results comprise the computed equilibrium paths and the LCO branches, presented using the $M_{y}$ response. The latter are depicted using the maxima and the minima of the LCO responses.

As shown, the resulting LCO solutions emerge and vanish at the corresponding Hopf bifurcations that subtend the regions of unstable equilibria. Further, as suggested by Fig. 8, the second Hopf bifurcation point tends to occur at the lower speeds with the higher pitch settings. This behaviour is also confirmed by the experimental results in Fig. 9. With the higher pitch setting in Fig. 9(a), the LCO amplitudes sharply drop on approaching the second Hopf Bifurcation. As opposed to this, with the lower pitch setting in Fig. 9(b), the experimental LCO labelled II only has a slightly smaller amplitude than the same recorded at the airspeed immediately before it. Note that the continued experimental testing towards the higher speeds was restricted for the safety reasons [33] and, hence, the second experimental Hopf bifurcation was only observed in the case where $\alpha_{{{\text{expr}}}} = 1.4^{ \circ }$. Furthermore, as suggested by the trends in the experimental results, no evidence of subcritical LCO behaviour was noted during testing (subject to the smallest airspeed increment/decrement that could be achieved reliably in the wind tunnel, when attempting to explore this about the flutter speed). Its absence is also reflected by the numerical results. This could be a result of the absence of aerodynamic stall. For instance, in [29], it was noted that a model without aerodynamic stall predicted supercritical LCOs, whereas the experiment indicated subcritical behaviour due to stalling.

As seen in the numerical LCO results, the higher amplitude LCO responses were identified as being unstable. The bifurcations between the stable and unstable portions of the LCO solutions were classified as the Neimark-Sacker bifurcations that marks the origin of quasi-periodic responses [47]. Owing to the experimental safety considerations, it cannot be conclusively confirmed whether the expected quasi-periodic behaviour reflects the reality because these parametric regions with large amplitude responses were not experimentally explored in sufficient detail. None of the experimental responses suggested such tendencies. The predicted behaviour can be a purely numerical artefact specific to the model arising from its limitations such as an ad-hoc applied structural damping model, simplified LLM aerodynamic formulation with ONERA-based locally 2D aerodynamics or truncation of the higher order nonlinear terms. Despite this, as evidenced by Fig. 9, the model is in reasonable agreement with the experimental behaviour at the lower amplitude LCOs near their emergence at the first Hopf Bifurcation, particularly in terms of the rate of growth of LCO amplitudes with airspeeds. The figure however suggests a growing discrepancy in the predictions and the LCOs as the solutions approach the second Hopf bifurcation, particularly at the lower of the two pitch setting studied here. This could be linked to the discrepancies in the equilibrium paths as discussed in Sect. 5.1, where the model could fail to locate the second Hopf Bifurcation due to the incorrect level of static deflection (and thus the growing discrepancy of the LCO solutions as they approach the second Hopf Bifurcation). On the other hand, this could also be linked to the unsteady aerodynamic representation and the tuning of its parameters to which the Hopf Bifurcations are highly sensitive. Essentially, with the highly sensitive instability thresholds associated with hump modes as recognised in [29], the behaviour of the LCO branches connecting them will reflect the same sensitivities to the aerodynamic modelling methods.

**Fig. 10:**
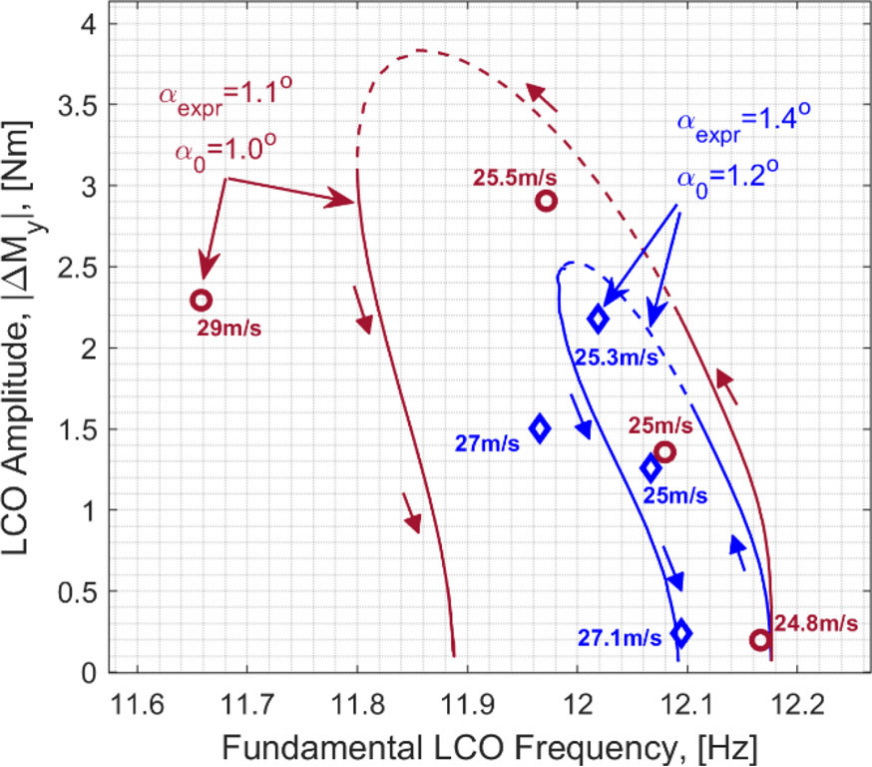
Fundamental LCO frequencies against the ‘peak-to-peak’ *M*_*y*_ amplitudes. The two cases correspond to the 1-parameter LCO trajectories in Fig. 9. The corresponding experimental results are indicated by the markers of the same colour

To extend the comparison to the frequency-amplitude features of the LCOs, Fig. 10 presents the relationship between the fundamental LCO frequencies and the amplitudes for the 1-parameter LCO trajectories shown in Fig. 9. The amplitude included on the vertical axis is the peak-to-peak *M*_*y*_ amplitude encountered during the LCO response with each parameter combination. Note that the colours used in this figure deviate from those defined in the key included in Fig. 7, as the two cases with the different wing root pitch settings are considered here.

As indicated by the figure, the model correctly captures the experimentally noted trend associated with the ‘loop-shaped’ frequency vs amplitude behaviour. With increasing *U*, the LCO frequency decreases with growing amplitudes across the initial phase of the LCO branch. Towards the final part of the LCO branch, when nearing the second Hopf bifurcation, the frequencies increase slightly as the amplitude drops.

**Fig. 11:**
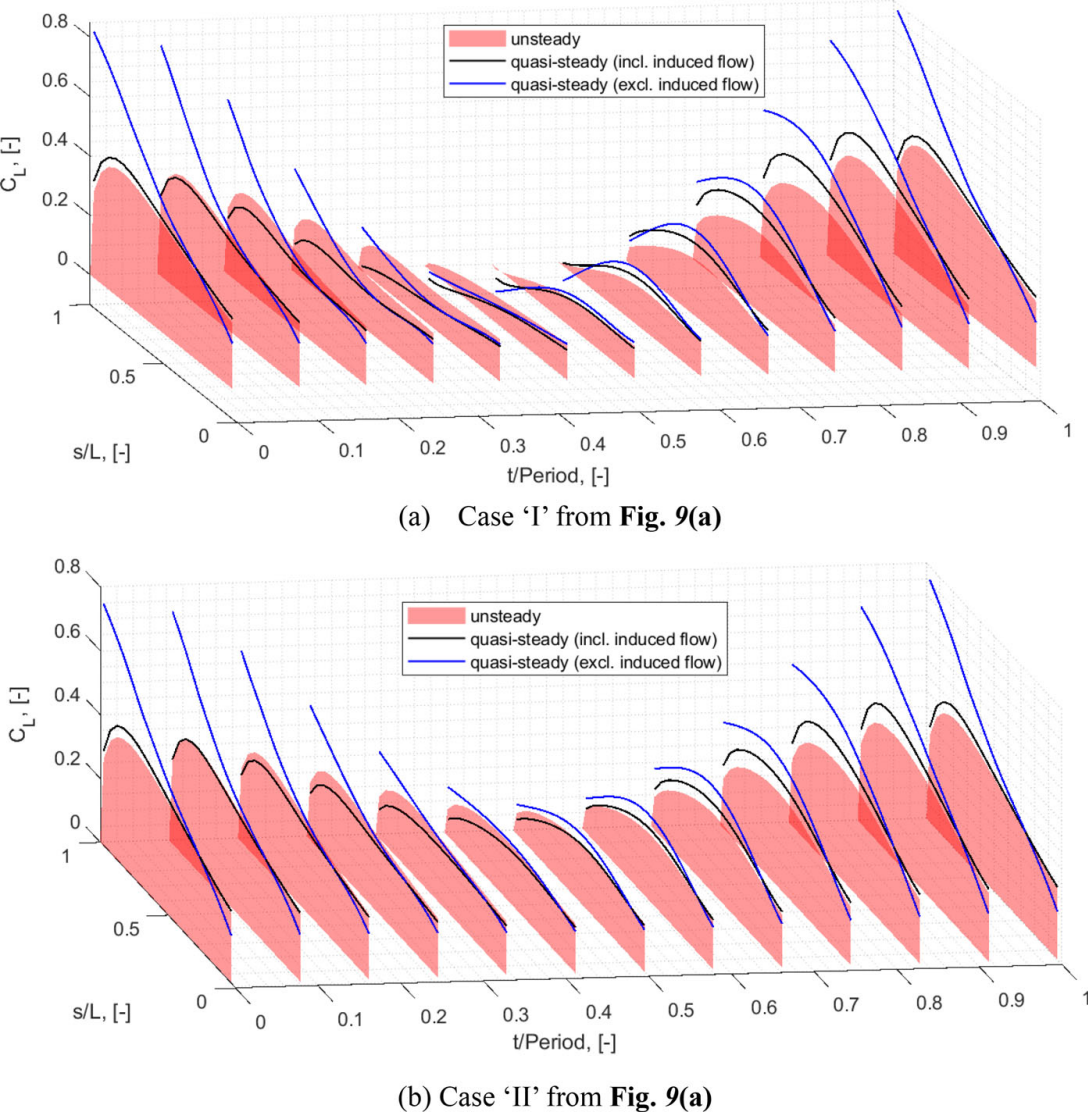
Comparison of the unsteady lift distributions against their quasi-steady equivalents during LCOs

Finally, to highlight the significance of the key feature of the developed model, namely the ability to take into account the finite span effects along with unsteady aerodynamics, Fig. 11 provides the ‘stroboscopic’ views of the spanwise lift distributions during LCO responses at selected parameter combinations. These are visualised for the cases identified as ‘I’ and ‘II’ in Fig. 9(a). The figure compares the lift distributions obtained under different assumptions to convey their significance,

- The *unsteady lift distribution*, ${\mathbf{C}}_{{\mathbf{L}}} (t)$, as implemented in the model and used in the computations.
- The *quasi-steady equivalent with the induced flow* (finite span effects included): These are simply computed from the steady $c_{l} (\alpha )$ functions, with the (spanwise) local angles of attacks derived using quarter-chord downwash, $W_{0}$, that includes the vortex downwash distribution, ${\mathbf{u}}_{{{\mathbf{ind}}}}$, derived from the ${{\varvec{\Gamma}}}(t)$ response.
- The *quasi-steady equivalent excluding the induced flow*: same as above, with the quarter-chord downwash, $W_{0}$, excluding the effects of ${\mathbf{u}}_{{{\mathbf{ind}}}}$ (that is, only the geometrical flow component included—see Sect. 3.4.2)

Firstly, a noteworthy observation in the unsteady lift distribution response is that the localised lift coefficients remain well within the attached flow range during the selected LCO instances shown above. This suggests that this range of the LCO responses is primarily driven by the (structural) geometrical nonlinearity.

Secondly, the behaviour of the complete unsteady response can be described as one that lags the quasi-steady response with the induced flow, with the latter exhibiting relatively comparable lift magnitudes despite the incorrect phase. The discrepancy between their phasing is particularly seen in Fig. 11(a), where the negative quasi-steady lift levels are observed near the wing tip (*t*/Period ~ 0.5). Finally, it is clear, across both cases, that an aerodynamic formulation that does not incorporate finite-span effects (induced flow) would result in a substantial error in the lift distribution, given the aspect ratio of the studied wing configuration.

## Aeroelastic modal analysis

The modal characteristics of the studied wing vary with airspeed. This section develops a comparison between the numerical model and experiments with a focus on these characteristics. The variations of the modal frequencies and the damping ratios with increasing airspeed are presented. The numerical modal coupling indicators in the critically behaved aeroelastic mode are also provided.

All the results in this section corresponds to the constant wing root pitch setting already presented in Fig. 9(a), where $\alpha_{{{\text{expr}}}} = 1.4^{ \circ }$ case is compared against the matched numerical case where $\alpha_{0} = 1.2^{ \circ }$. This specific case is selected given the availability of the equilibrium experimental test points that follow the airspeed-bounded unstable zone, allowing the chosen modal characteristics to be compared across both the pre- and post-flutter conditions. The numerical modal characteristics are extracted using the linearised state-space model established numerically by performing small perturbations about the continuation-based equilibria. The experimental results were using the Operational Modal Analysis (OMA) framework described in [33].

### Evolutionary modal characteristics

**Fig. 12:**
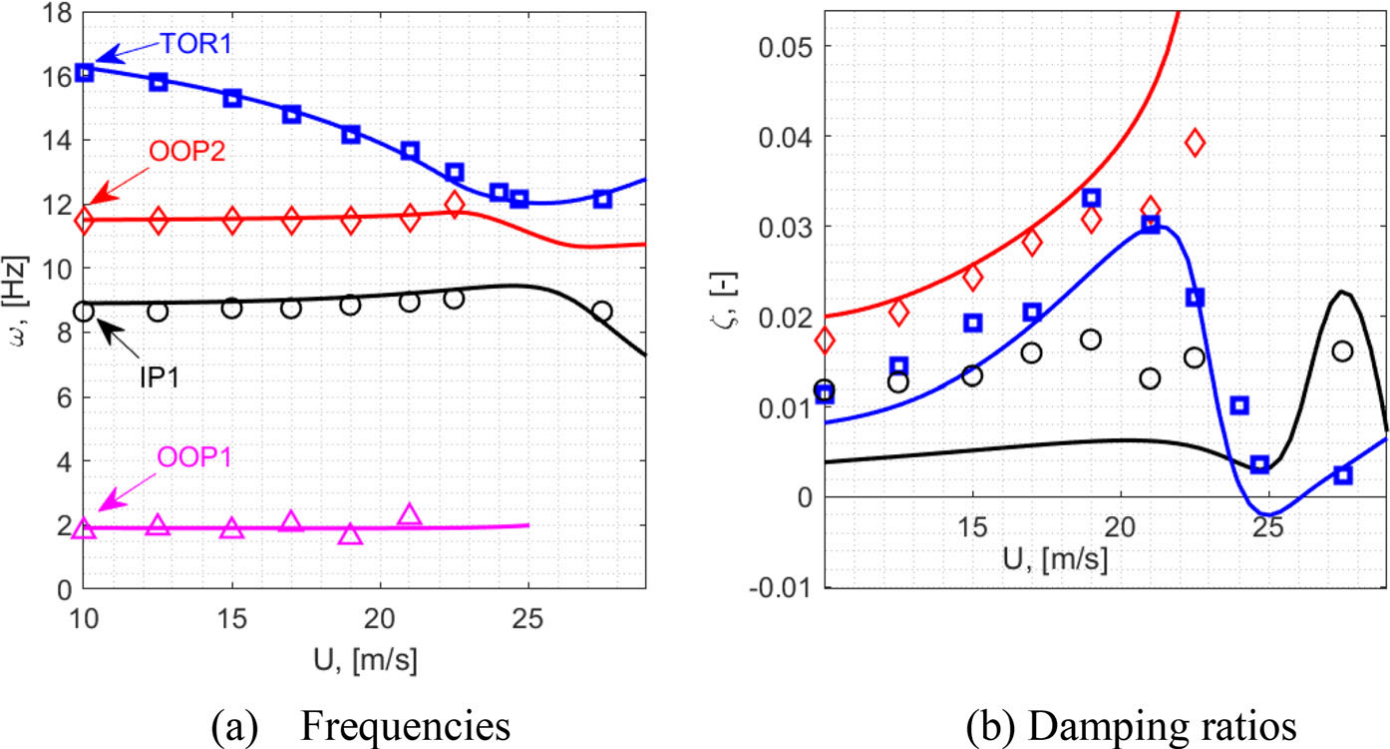
Airspeed dependent variations of the modal frequencies and damping ratios corresponding to the equilibria in Fig. 9(a). Curves—numerical results. Markers—experimental results. Note that positive damping ratios indicate a stable mode

Figure 12 presents the frequency and damping variations observed when increasing airspeed. The first four aeroelastic modes are examined. They comprise the modal loci originating as the first two out of plane bending modes (OOP1–OOP2), the first in-plane bending mode (IP1) and the first torsional mode (TOR1).

As evidenced by the figure, a good match for both the frequency and damping loci is obtained between the model and experiment. This includes the key features exhibited by the critical modal loci originating as the TOR1 mode (extensively discussed in [33]), namely, the progressive drop of its frequency towards the OOP2 locus with increasing *U* and the ‘hump’ feature of its damping ratio that ventures into the negative values, indicating instability.

To extend this comparative study, a previously proposed quantitative indicator of modal coupling [33] is adopted here to elucidate and compare the evolution of the modal participation for the model and the corresponding experiments. As discussed in Sect. 4.3, the structural motions were described using a series of modal coordinates, ${{\varvec{\upgamma}}}(t)$, which were transformed to the generalised coordinates through the reference mass-normalised eigenmode basis ${\mathbf{\varphi }} \in {\mathbb{R}}^{N \times N}$. As detailed previously, the reference modal basis used for this process was obtained about the equilibrium under the self-weight of the wing.

After solving the aeroelastic eigenvalue problem at each speed, the structural components of the aeroelastic eigenvectors, ${\hat{\mathbf{\gamma }}}^{( \bullet )} \in {\mathbb{C}}^{N \times 1}$, are extracted where the superscript identifies each aeroelastic mode. This eigenvector is essentially a projection of the structural component of the aeroelastic eigenvector to the reference modal basis. As such, this quantity is indicative of the coupled contributions from the reference modal basis, ${\mathbf{\varphi }}$ (as defined in Sect. 4.3) in the evolved aeroelastic eigenvectors. This important perspective is used in the following analysis to focus the study to show that the crucial modal interactions are correctly predicted by the developed model via comparison with the equivalent experimental information.

Given the two-degree-of-freedom flutter mechanism observed in this study, and as extensively discussed in [33], the critical aeroelastic eigenvector, ${\hat{\mathbf{\gamma }}}^{{{\text{TOR1}}}}$, is known to be dominated by the reference TOR1 and OOP2 components. The corresponding vector elements are the complex-valued scalars denoted ${\hat{\mathbf{\gamma }}}_{{{\text{TOR1}}}}^{{{\text{TOR1}}}}$ and ${\hat{\mathbf{\gamma }}}_{{{\text{OOP2}}}}^{{{\text{TOR1}}}}$, where the subscript identifies the specific element of the ${\hat{\mathbf{\gamma }}}^{{{\text{TOR1}}}}$ aeroelastic eigenvector. The comparison that follows concerns the relative participation between these two reference components in the critical aeroelastic mode ${\hat{\mathbf{\gamma }}}^{{{\text{TOR1}}}}$. In particular, the argument between ${\hat{\mathbf{\gamma }}}_{{{\text{TOR1}}}}^{{{\text{TOR1}}}}$ and ${\hat{\mathbf{\gamma }}}_{{{\text{OOP2}}}}^{{{\text{TOR1}}}}$ is indicative of the phase lag between the reference TOR1 component (indicative of torsional motions) and the reference OOP2 component (indicative of bending motions) in the coupled aeroelastic eigenvector. The magnitude ratio between the same indicates the relative participation amplitude. This approach provides a quantitative, reduced form of comparison against the experimental counterpart as further validation of the model-based, coupled bending-torsion response content of the critically behaved aeroelastic mode.

The experimental counterpart of the above process, as described in [33], was developed with mode shapes represented in terms of the sensor participation factors. That is, the reference modal basis was generated from (wind off) experimental modal analysis, with the eigenvectors extracted in the form of a vector of sensor-participation factors for the combination of strain instrumentation and the accelerometer. The aeroelastic eigenvectors were established in the same format, using operational modal analysis. Then, the aeroelastic eigenvectors are projected on to the experimental reference basis, allowing them to be described using complex participation factors that scale the reference modes. The underlying procedure is extensively described in [33]. From this, the same relative magnitude and phase information between the reference TOR1 and OOP2 participation factors in the critical aeroelastic mode can be established for comparison.

Due to the above sensor-based approach in the experimental counterpart, the experimental mode shapes in [33] were normalised with measurement-based criteria: The TOR1 and the OOP2 vectors were normalised to achieve a unit participation on the torsional moment and the root shear (in kg) measurements respectively. As such, for the model-based counterpart, in lieu of comparing the complex argument and magnitude of the ratio, ${\hat{\mathbf{\gamma }}}_{{{\text{OOP2}}}}^{{{\text{TOR1}}}} /{\hat{\mathbf{\gamma }}}_{{{\text{TOR1}}}}^{{{\text{TOR1}}}}$, a scaled version of the same is considered in the form,

45 $$ \beta_{{{\text{OOP2}}}}^{{{\text{TOR1}}}} = \frac{{f_{{{\text{OOP2}}}} }}{{f_{{{\text{TOR1}}}} }}\left( {\frac{{{\hat{\mathbf{\gamma }}}_{{{\text{OOP2}}}}^{{{\text{TOR1}}}} }}{{{\hat{\mathbf{\gamma }}}_{{{\text{TOR1}}}}^{{{\text{TOR1}}}} }}} \right) $$

which preserves the effects of the normalisations used in the experiment-based approach. In the above, the terms *f*_*()*_ are the factors used to introduce a re-scaling of the mass normalised reference basis. These scaling factors take the form,

46 $$ f_{{{\text{OOP2}}}} = \frac{1}{{{\mathbf{V}}_{Sz}^{{}} {\mathbf{\varphi }}^{{{\text{OOP2}}}} }},\quad f_{{{\text{TOR1}}}} = \frac{g}{{{\mathbf{V}}_{Mx}^{{}} {\mathbf{\varphi }}^{{{\text{TOR1}}}} }} $$

where ${\mathbf{V}}_{Sz}^{{}}$ and ${\mathbf{V}}_{Mx}^{{}}$ are as defined in Eq. (40), ${\mathbf{\varphi }}^{{{\text{OOP2}}}}$ and ${\mathbf{\varphi }}^{{{\text{TOR1}}}}$ are the reference OOP2 and the TOR1 vectors from the reference basis (from Sect. 4.3) and *g* = 9.81 m/s^2^ is the gravitational acceleration.

**Fig. 13:**
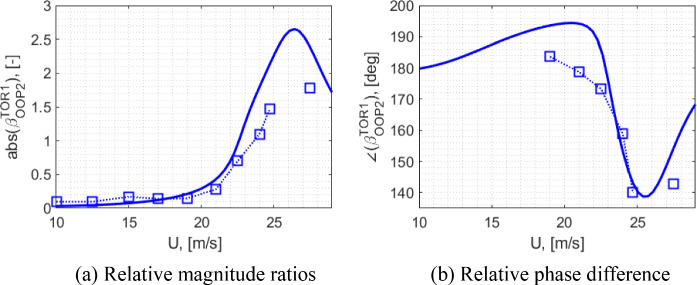
Relative participation features of the wind-off TOR1 and OOP2 components on the coupled critical aeroelastic mode as determined by means of the proposed model (solid lines) and the experimental data (square markers)

Figure 13 compares the relative phase and magnitude of this quantity between the model and the experimentally derived values, providing the previously discussed relative phase-lag and amplitude information of the coupled representative bending-torsion content of the critical aeroelastic mode.

The behaviour suggested in the figure, particularly in Fig. 13(a), indicates the expected behaviour with $\beta_{{{\text{OOP2}}}}^{{{\text{TOR1}}}}$ tending towards zero on approaching wind off conditions (*U* = 0). With increasing flow speeds, the magnitude of $\beta_{{{\text{OOP2}}}}^{{{\text{TOR1}}}}$ progressively grows, indicating the intense coupling between the bending and the torsional components. Overall, the modal composition predicted numerically can be seen to be in good agreement with the experimental results. In particular, the progressively increasing participation of the reference OOP2 component, and the ~ 140-degree phase lag feature of this component relative to the reference TOR1 component in the critical mode is seen in both cases. This clearly suggests that the destabilising mode in the model comprises of the same coupled complex participation content as the experiment.

### Sensitivity to unsteady aerodynamic characteristics

As highlighted in Sect. 3.4, the aerodynamic formulation adopted in this modelling method consists of a series of semi-empirical coefficients that vary between different aerofoils and test conditions (e.g. Reynold’s number). It is recognised that these coefficients affect solely the dynamic characteristics of the unsteady lift responses, and not the static behaviour. This section examines the sensitivity of the aeroelastic modal characteristics to changes of the ONERA unsteady coefficients $\lambda_{L}$ and $\alpha_{L}$ from Eq. (22). This is examined sequentially, first introducing the relationship between the coefficients in question and the flutter speed itself, and then developing towards the underlying sensitivities of the modal damping ratios that dictates the flutter speed. Ultimately, the underlying unsteady lift response behaviour is interpreted in frequency domain, and some similarities between the noted behaviour and that under aerodynamic effects (particularly Reynold’s number) from literature are drawn.

**Fig. 14:**
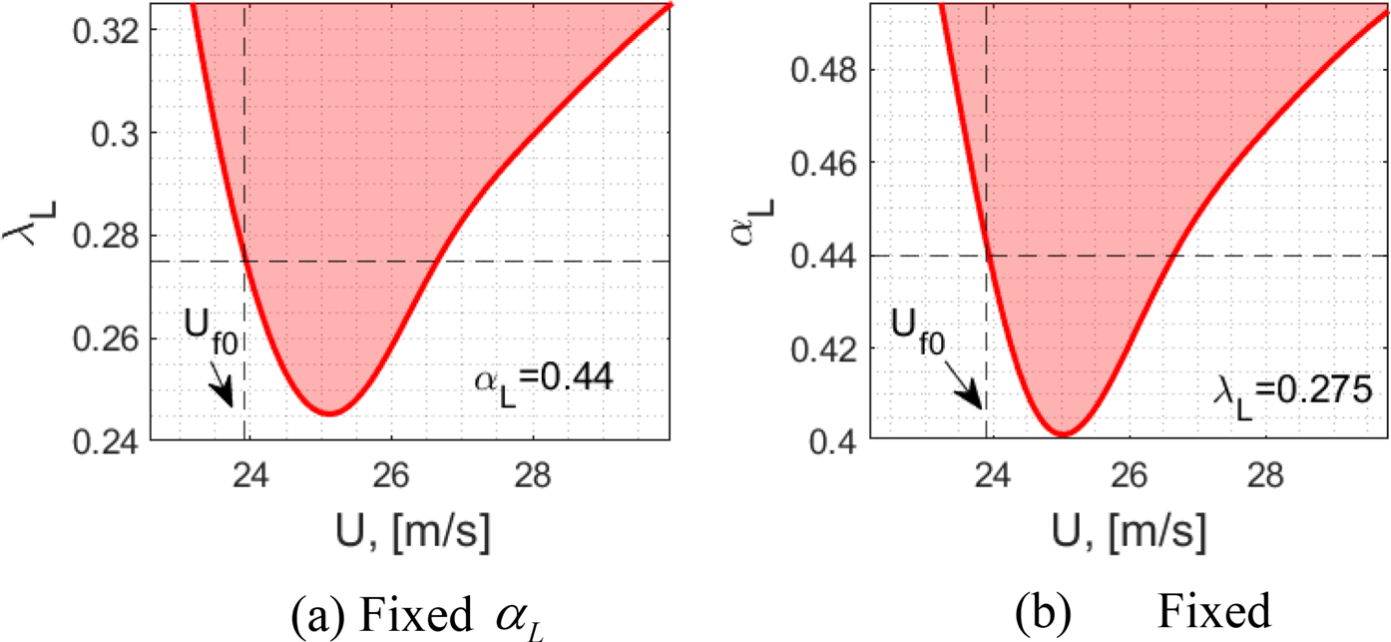
Effect of $\lambda_{L}$ and $\alpha_{L}$ on the instability boundary. Solid line—Hopf bifurcation boundary. Shaded region—unstable equilibria

To demonstrate these sensitivities systematically, first the sensitivity of the flutter speed itself is presented in Fig. 14, with each of the subfigures showing the effect of changing one coefficient whilst holding the other fixed. The original flutter speed (with the selected combination of $\lambda_{L}$ and $\alpha_{L}$) is denoted *U*_*f0*_. These results are generated through the 2-parameter numerical continuation traversing the Hopf bifurcations.

As illustrated by the figure, these unsteady coefficients influence the range of *U* across which the equilibria are unstable, with the flutter mechanism effectively ceasing to exist below a critical value of the unsteady parameters.

**Fig. 15:**
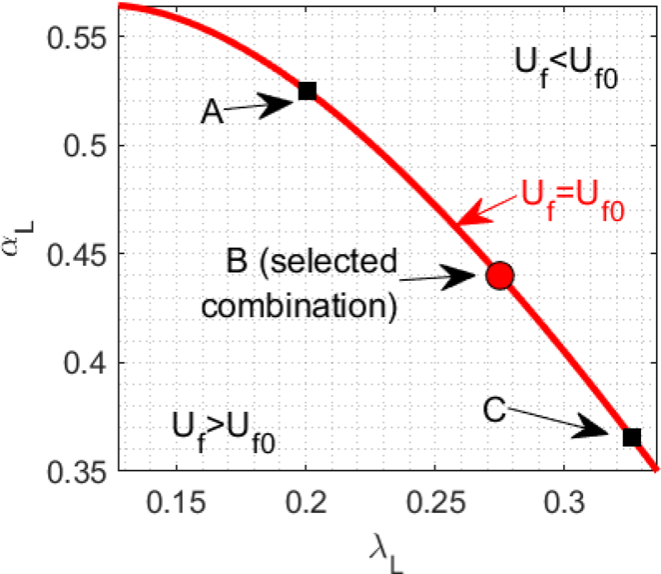
Combinations of $\lambda_{L}$ and $\alpha_{L}$ to preserve the original flutter speed *U*_*f0*_

Next, the relationship between $\lambda_{L}$ and $\alpha_{L}$ required to retain the flutter speed at *U*_*f0*_ is identified in Fig. 15. From this, the three cases identified as A, B and C are selected for the detailed examination, with case B being the parameter combination previously selected for the tuned model.

**Fig. 16:**
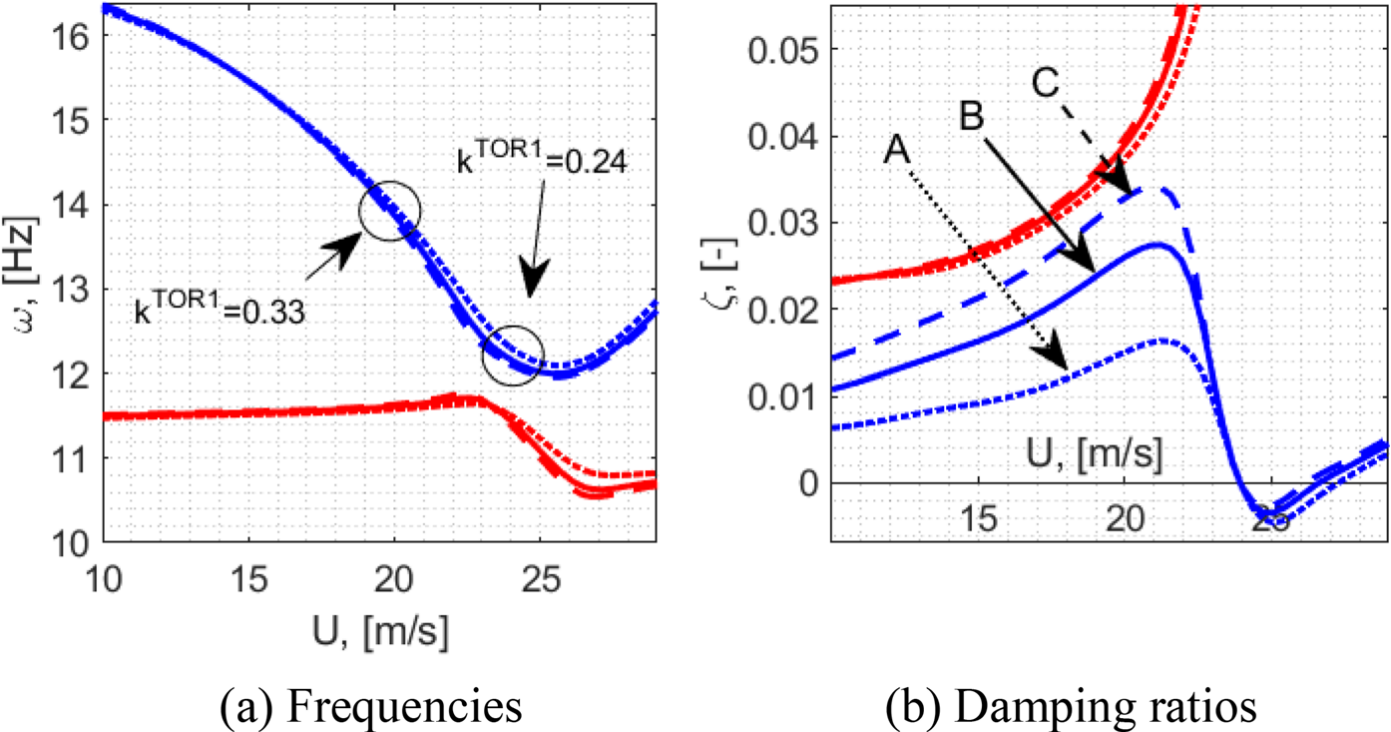
Variations of the frequency-damping loci of the OOP2 and TOR1 modes with different unsteady coefficient combinations from Fig. 15 (dotted-A, solid-B, dashed-C)

Figure 16 demonstrates the behaviour of the aeroelastic modes (emerging as the TOR1 and the OOP2) under the three conditions A-C identified above. As the flutter speed is preserved, the first intercept of the zero-damping ratio threshold is obtained at the same speed.

As illustrated by the figure, the most prominent effects are observed on the damping ratios of the critical aeroelastic mode (originating as the TOR1 mode), where the pre-flutter damping ratios are significantly altered. Noting this sensitivity, the selected ONERA parameters (case ‘B’) used to develop the experimental comparisons was informed by the experimental modal damping ratios in conjunction with the flutter onset speed.

To provide a deeper appreciation of how the studied unsteady coefficients drive this behaviour, their effects on the frequency-domain response of the lift coefficient to the angle of attack, $\alpha$, inputs created by pitching oscillations are considered. This is represented as a frequency domain output-input ratio (transfer function) referred to as the *lift deficiency function*. Based on Eq. (22), this can be written as [40],

47 $$ {\text{Lift}}\;{\text{Deficiency}} = \frac{{C_{L} (k)}}{{c_{{l_{\alpha } }} \alpha (k)}} = \frac{{\lambda_{L} + {\text{i}}k\alpha_{L} }}{{\lambda_{L} + {\text{i}}k}} $$

In the above, $k = \omega b/U$ is the reduced frequency. This is a representation of the frequency based on the time taken by the flow to pass through a semi-chord. For a fixed modal frequency, the reduced frequency drops with increasing speeds. The reduced frequency of the critical mode at two selected airspeeds are marked in Fig. 16(a).

**Fig. 17:**
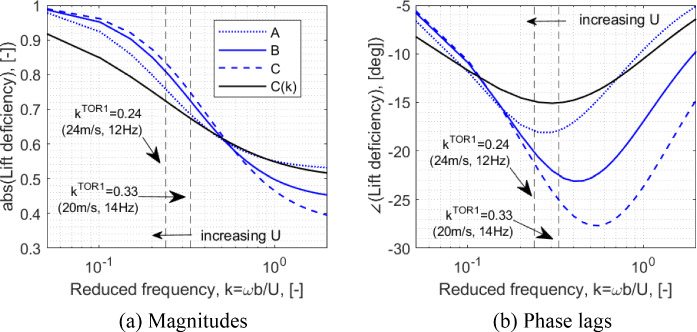
Behaviour of the lift deficiency function (in the frequency domain) with different unsteady parameter combinations from Fig. 15

Figure 17 identifies the magnitude and the phase lag features of the lift deficiency functions obtained with the three parameter combinations A–C. Additionally, the Theodorsen-based equivalent referred to as the *C(k)* function [48] is also compared as a representation of the theoretical flat-plate and inviscid result.

The difference in the magnitudes of the lift deficiency function between the different cases drops with initially increasing reduced frequencies below *k* ~ 0.5, whilst the phase lag is increased between cases A to C.

A particular emphasis is dedicated to the frequencies marked with the vertical dashed lines that corresponds to the indicated frequencies in Fig. 16(a) along the critical mode. Noting that *k* increases when reducing *U* from *U*_*f0*_, the more substantial changes to the phase lag in the lift deficiency function can be suggested as the feature that drives the damping ratio sensitivities at pre-flutter speeds (as the difference in the magnitudes only reduce between A and C when *U* is reduced from the flutter speed).

The behaviour associated with the phase-lag has been recognised to originate due to viscosity related factors [49]. In particular, lower Reynold’s numbers have been shown to induce larger phase lags in comparison to the Theodorsen’s *C(k)* function. The phase lag of the lift deficiency function with the tuned ONERA coefficients shows a behaviour that is consistent with this when compared against the Theodorsen function.

The selective sensitivity of modal damping to this phase lag can, to some extent, be associated with the intricate phasing features between the pitching/heaving displacements and the aerodynamic forcing. That is, under pure pitching and plunging motions, the angles of attack are largely driven by the isolated plunging velocity and the pitching displacement, respectively. However, the work done is a combined function of these quantities and the respective displacements in each case. As the phase features of the lift deficiency function further influence these intricate relationships, they can have selective influences on the modes depending on their coupled bending and torsional content. The phase offset originating from the lift deficiency function introduces an additional offset to the loading component that can impact the work done per cycle when combined with the corresponding displacement.

As suggested by these observations, the flow-dependent unsteady aerodynamic behaviour, which in the model is driven by the examined ONERA coefficients, can substantially influence the modal characteristics of the system and the flutter onset itself. In particular, it is recognised that the Reynold’s number dependency of the unsteady lift behaviour likely had a substantial influence on the damping behaviour of the critical aeroelastic mode. Hence, whilst the present comparisons were generated with fixed (representative) unsteady parameters used across all the studied airspeeds, it could be more appropriate to implement the airspeed dependency of these parameters for refined application of the model. All in all, application of the developed model for predictive purposes requires information of carefully characterised unsteady parameters for the model. Where such application-specific information is not available, the model-based interpretation of predicted dynamics requires an appreciation of their sensitivities to variations of the unsteady parameters within a stipulated range, as demonstrated. This is imperative to exploit the predictive capability of the developed low order framework that was evidenced by the presented comprehensive experimental comparisons.

## Conclusions

This paper develops and validates a mathematical model of a flexible cantilevered wing, with a novel approach to capture the unsteady aerodynamic loads with the finite-span effects. The geometrically nonlinear structural model is developed using a Chebyshev–Ritz approach, with the out-of-plane (chordwise) and in-plane bending and the torsional deflections discretised with the appropriate shape functions. The aerodynamic model combines the formulation of the circulatory lift coefficient from the ONERA model with the classical lifting line-based description of the circulation distribution across the spanwise strips. The resulting nonlinear system is assembled in the state space form, with the states comprising of the structural coordinates, velocities and the aerodynamic states.

The developed model is substantiated and validated using the comprehensive experimental results obtained from the testing of a flexible cantilevered wing at the University of Bristol. The model-based numerical results for these comparisons are generated using the numerical continuation approach to systematically examine the system’s bifurcation environment.

The model was shown to possess a reliable capability to replicate experimentally characterised behaviours, but required appropriately adapted parameters for the ONERA unsteady aerodynamic sub-model. In particular, the model was able to capture the experimentally observed bifurcation features of the system, including the airspeed-bounded flutter instability, its migration with the changing deflection levels and the trends associated with the LCO responses and their orbits as they first emerge about flutter onset. The significance of the modelled spanwise unsteady lift distribution was studied with selected LCO orbits. It was shown that the quasi-steady equivalents and the absence of the finite-span effects (induced downwash) would yield notably different lift distributions subject to the same computed structural motions during the LCOs.

The comparisons were further extended to the airspeed-driven aeroelastic modal properties, with the model offering predictions in good qualitative and quantitative agreement across a wide range of conditions in terms of the natural frequencies and the damping ratios of the first four modes. Particularly, this included the frequency convergence behaviour of the modes contributing to the instability (second bending and first torsion), and the hump-shaped feature in the damping ratio trends of the critical modal locus (originating as the first torsion mode) responsible for instability. This comparative analysis was extended by recreating an experimentally implemented modal-projection approach where the coupled aeroelastic modes were expressed using a reference modal basis from wind-off conditions. This was used to show that the model was able to predict the evolution of the participation between the reference bending and torsional modal motions when assigned to the critical mode.

It was further shown that the pre-flutter damping ratios of the critical modal locus were highly sensitive to the ONERA unsteady coefficients. An extended analysis of the frequency domain behaviour of the ONERA-based lift coefficients suggested that this was associated mainly with the phase lag between the driving angle of attack inputs and the lift coefficient output. This sensitive phase lag behaviour was recognised to be consistent with previously published observations regarding the low Reynold’s number viscous effects.

Overall, the developed low order model, with the ability to represent the complex interactional physics of the aerodynamically loaded structure, was shown to possess a very good predictive capability when compared to the experimentally results, including the intricate and varying features of the aeroelastic modal characteristics. The primary challenge of this modelling method is the need for the identification of suitable unsteady aerodynamic modelling parameters. However, with its versatility arising from the compact and scalable state space differential form, it can be effectively used to interpret the underlying behaviours and trends in the tested systems, and to extrapolate analysis to the system with aeroelastic control augmentation.

## Figures and Tables

**Fig. 18:**
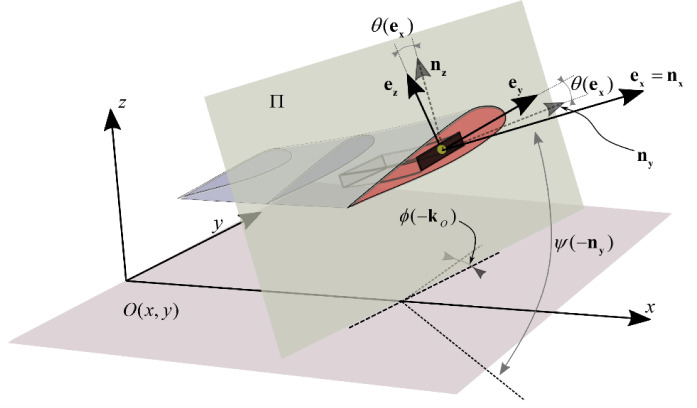
Angular rotations used to describe the deformed orientation of the local cross-sections

**Fig. 19:**
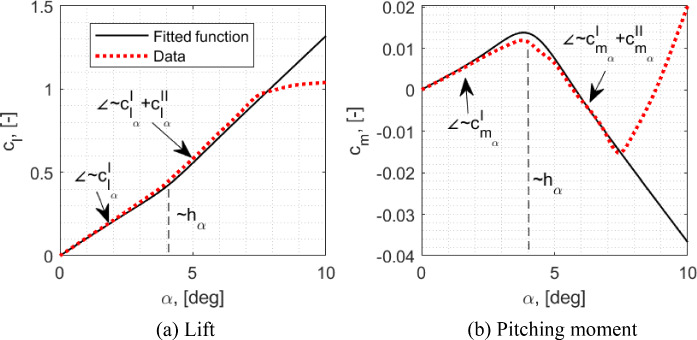
Aerodynamic coefficients at 25 m/s

**Fig. 20:**
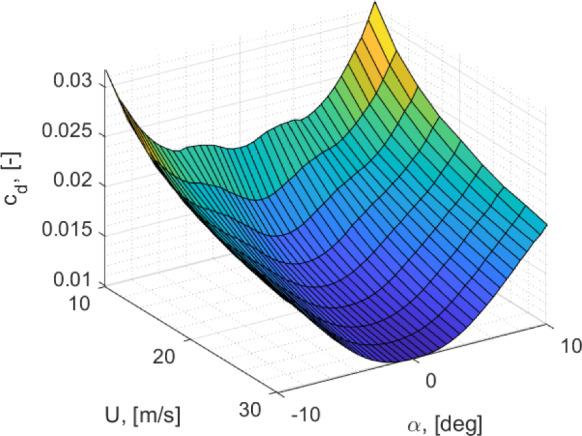
Drag coefficient data

## Data Availability

The data generated during the current study are available from the corresponding author on request.

## A. Nonlinear geometry

Figure 18 details the geometrical rotations used to describe the orientation of a local cross-section. Note that all angular definitions are presented with vector about which the angles are measured. Following from the definitions in Sect. 3.1, the axes local to the cross section are obtained as follows:

The plane of the local cross section $\Pi$, is assumed to remain normal to the locus of the Elastic Axis (EA). An intermediate frame $A$ described by the orthonormal triad ${\mathbf{n}}_{{\mathbf{x}}} ,{\mathbf{n}}_{{\mathbf{y}}} ,{\mathbf{n}}_{{\mathbf{z}}}$ is defined such that its origin coincides with the EA and ${\mathbf{n}}_{{\mathbf{x}}}$ is tangent to the locus of the EA and hence identical to the material axis ${\mathbf{e}}_{{\mathbf{x}}}$. Vectors ${\mathbf{n}}_{{\mathbf{y}}} ,{\mathbf{n}}_{{\mathbf{z}}}$ span $\Pi$ with ${\mathbf{n}}_{{\mathbf{y}}}$ being parallel to the line of intersection between the $O(x,y)$ plane and $\Pi$. The material-fixed frame $B$ given by the orthonormal triad ${\mathbf{e}}_{{\mathbf{x}}} ,{\mathbf{e}}_{{\mathbf{y}}} ,{\mathbf{e}}_{{\mathbf{z}}}$ is then obtained following a rotation by an angle $\theta (s,t)$, about the ${\mathbf{n}}_{{\mathbf{x}}}$ axis.

Accordingly, the angular definitions from Fig. 18 are described as follows: angle $\psi$ defines the smallest angle between the ${\mathbf{e}}_{{\mathbf{x}}}$ axis and the $O(x,y)$ plane, such that,

48 $$ \sin \psi = {\mathbf{k}}_{O}^{{\text{T}}} {\mathbf{e}}_{{\mathbf{x}}} = w^{\prime} $$

The angle $\phi$ defines the angle between the $O(y)$ axis (${\mathbf{j}}_{O}$), and the line of intersection between the planes $O(x,y)$ and $\Pi$. Hence, given the definition of ${\mathbf{n}}_{{\mathbf{y}}}$,

49 $$ {\mathbf{n}}_{{\mathbf{y}}} = \left[ {\begin{array}{*{20}c} {\sin \phi } & {\cos \phi } & 0 \\ \end{array} } \right]^{{\text{T}}} $$

Note that both $\varphi$ and $\phi$ are described implicitly as functions of $w^{\prime}$ and $v^{\prime}$, in contrast to the explicitly defined torsional angle $\theta (s,t)$ used to define the transition between the frame $A$ and the material frame $B$ as detailed above.

Using the above definitions, the angles $\varphi$ and $\phi$ can be derived as follows. Following from Eq. (48), applying the Taylor approximation to the inverse of sine ratio yields,

50 $$ \psi \approx w^{\prime} + \frac{1}{6}w^{{\prime}{3}} $$

Considering the orthogonality between ${\mathbf{n}}_{{\mathbf{x}}} ( = {\mathbf{e}}_{{\mathbf{x}}} )$ and ${\mathbf{n}}_{{\mathbf{y}}}$, ${\mathbf{n}}_{{\mathbf{x}}}^{{\text{T}}} {\mathbf{n}}_{{\mathbf{y}}} = 0$, along with Eq. (49) can be used to obtain,

51 $$ (1 + u^{\prime})\sin \phi + v^{\prime}\cos \phi = 0 $$

which, along with Eq. (3) can be used to derive,

52 $$ \sin \phi \approx - v^{\prime}\left( {1 + \frac{1}{2}w^{{\prime}{2}} } \right),\quad \cos \phi \approx 1 - \frac{1}{2}v^{{\prime}{2}} \left( {1 + w^{{\prime}{2}} } \right) - \frac{1}{8}v^{{\prime}{4}} $$

and hence,

53 $$ \phi \approx - v^{\prime}\left( {1 + \frac{1}{2}w^{{\prime}{2}} } \right) - \frac{1}{6}v^{{\prime}{3}} $$

With the angles and their trigonometric ratios derived implicitly, the derivation of the axes of frames and can be completed. The axis ${\mathbf{n}}_{{\mathbf{y}}}$ is derived as per Eq. (49) applied with Eq. (52). Axis ${\mathbf{n}}_{{\mathbf{z}}}$ then follows naturally from,

54 $$ {\mathbf{n}}_{{\mathbf{z}}} = {\mathbf{n}}_{{\mathbf{x}}} \times {\mathbf{n}}_{{\mathbf{y}}} $$

The axes of frame $B$ can be derived using the above results and can be written as follows:

55 $$ \begin{aligned} {\mathbf{e}}_{{\mathbf{x}}} & = \frac{{{\mathbf{r^{\prime}}}_{{{\text{EA}}}} (s,t)}}{{\left\| {{\mathbf{r^{\prime}}}_{{{\text{EA}}}} (s,t)} \right\|}} = \left[ {\begin{array}{*{20}c} {1 + u^{\prime}} & {v^{\prime}} & {w^{\prime}} \\ \end{array} } \right]^{{\text{T}}} \\ & \approx \left[ {\begin{array}{*{20}c} {1 - \frac{1}{2}\left( {w^{{\prime}{2}} + v^{{\prime}{2}} } \right) - \frac{1}{8}\left( {w^{{\prime}{4}} + v^{{\prime}{4}} + 2w^{{\prime}{2}} v^{{\prime}{2}} } \right),} & {v^{\prime},} & {w^{\prime}} \\ \end{array} } \right]^{{\text{T}}} \\ {\mathbf{e}}_{{\mathbf{y}}} & = {\mathbf{n}}_{{\mathbf{y}}} \cos \theta + {\mathbf{n}}_{z} \sin \theta \\ & \approx \left[ {\begin{array}{*{20}c} { - v^{\prime}\left( {1 - \frac{1}{2}\theta^{2} + \frac{1}{2}w^{{\prime}{2}} } \right) - w^{\prime}\theta ,} & {1 - \frac{1}{2}\theta^{2} - \frac{1}{2}v^{{\prime}{2}} - w^{\prime}\theta v^{\prime},} & {\theta \left( {1 - \frac{1}{2}w^{{\prime}{2}} } \right) - \frac{1}{6}\theta^{3} } \\ \end{array} } \right]^{{\text{T}}} \\ {\mathbf{e}}_{{\mathbf{z}}} & = {\mathbf{n}}_{{\mathbf{z}}} \cos \theta - {\mathbf{n}}_{{\mathbf{y}}} \sin \theta \\ & \approx \left[ {\begin{array}{*{20}c} { - w^{\prime}\left( {1 - \frac{1}{2}\theta^{2} - \frac{1}{2}v^{{\prime}{2}} } \right) + v^{\prime}\theta ,} & {\frac{1}{6}\theta^{3} - \theta \left( {1 - \frac{1}{2}v^{{\prime}{2}} } \right) - w^{\prime}v^{\prime},} & {1 - \frac{1}{2}\theta^{2} - \frac{1}{2}w^{{\prime}{2}} } \\ \end{array} } \right]^{{\text{T}}} \\ \end{aligned} $$

which as shown above, can ultimately be written as Taylor-expanded polynomials of the three translational and the torsional degrees of freedom used to describe the problem.

Finally, the angular velocity terms can be developed based on the projections of the angular definitions as shown in Fig. 18. In the context of the kinetic energy formulations presented in “Appendix B”, it is useful to develop the angular velocity vector in the material frame $B$, as the components of the inertia tensor are known in this frame. The projection of the angular velocity components on the orthonormal triad of this frame thus takes the form:

56 $$ {{\varvec{\Omega}}} = \left[ {\begin{array}{*{20}l} { - \dot{\phi }\sin \psi + \dot{\theta }} \\ { - \dot{\phi }\cos \psi \sin \theta - \dot{\psi }\cos \theta } \\ { - \dot{\phi }\cos \psi \cos \theta + \dot{\psi }\sin \theta } \\ \end{array} } \right]_{B} \approx \left[ {\begin{array}{*{20}c} {\dot{v}^{\prime}w^{\prime} + \dot{\theta }} \\ {\dot{v}^{\prime}\theta - \dot{w}^{\prime}\left( {1 - \frac{1}{2}\theta^{2} + \frac{1}{2}w^{{\prime}{2}} } \right)} \\ {\dot{v}^{\prime}\left( {1 + \frac{1}{2}v^{{\prime}{2}} - \frac{1}{2}\theta^{2} } \right) + \dot{w}^{\prime}\left( {w^{\prime}v^{\prime} + \theta } \right)} \\ \end{array} } \right]_{B} $$

which, as shown above, can once again be written using Taylor-expanded polynomials using the results of Eqs. (50) and (53) that of the trigonometric ratios seen in the exact expressions.

## B. Structural energy formulations

The formulations for the structural potential energy and the structural kinetic energy used to develop the Lagrangian structural equations in Sect. 3.3 are presented here. These are developed utilising the geometrical definitions presented in Sect. 3.1, and the nonlinear geometrical relationships from “Appendix A”.

The structural potential energy U is first developed by adopting the Green–Lagrange (G–L) strain formulation. Consider a general point on the undeformed beam, ${\mathbf{r}}^{{{\text{undef}}}}$, as described in Eq. (5), and a second point on the same at an incremental offset ${\mathbf{r}}^{{{\text{undef}}}} + {\text{d}}{\mathbf{r}}^{{{\text{undef}}}}$. Suppose that the incremental vector joining the two points, upon deformation, becomes ${\text{d}}{\mathbf{r}}$. These incremental vectors before and after deformation can be compiled as,

57 $$ \begin{aligned} {\text{d}}{\mathbf{r}}^{{{\text{undef}}}} & = \left[ {\begin{array}{*{20}c} {{\text{d}}s} & {{\text{d}}n_{y} } & {{\text{d}}n_{z} } \\ \end{array} } \right]^{{\text{T}}} = {\mathbf{g}}_{x} {\text{d}}s + {\mathbf{g}}_{y} {\text{d}}n_{y} + {\mathbf{g}}_{z} {\text{d}}n_{z} \\ {\text{d}}{\mathbf{r}} & = {\mathbf{r^{\prime}}}{\text{d}}s + {\mathbf{e}}_{{\mathbf{y}}} {\text{d}}n_{y} + {\mathbf{e}}_{{\mathbf{z}}} {\text{d}}n_{z} = {\mathbf{G}}_{x} {\text{d}}s + {\mathbf{G}}_{y} {\text{d}}n_{y} + {\mathbf{G}}_{z} {\text{d}}n_{z} \\ \end{aligned} $$

The outer products of ${\mathbf{g}}_{i} ,{\mathbf{G}}_{i}$($i \in \left\{ {x,y,z} \right\}$) can be used to compile the terms in the G–L strain tensor as [50],

58 $$ \tilde{\varepsilon }_{ij} = \frac{1}{2}\left( {{\mathbf{G}}_{i}^{{\text{T}}} {\mathbf{G}}_{j} - {\mathbf{g}}_{i}^{{\text{T}}} {\mathbf{g}}_{j} } \right),\quad i,j \in \left\{ {x,y,z} \right\} $$

Under the assumption of small strains, the G–L strains are considered equivalent to the Engineering strains so that the required strains can be established as,

59 $$ \begin{aligned} \varepsilon_{xx} & \approx \tilde{\varepsilon }_{xx} = \frac{1}{2}\left( {{\mathbf{r^{\prime}}}^{{\text{T}}} {\mathbf{r^{\prime}}} - 1} \right), \\ \gamma_{xy} & \approx 2\tilde{\varepsilon }_{xy} = {\mathbf{r^{\prime}}}^{{\text{T}}} {\mathbf{e}}_{{\mathbf{y}}} , \\ \gamma_{xz} & \approx 2\tilde{\varepsilon }_{xz} = {\mathbf{r^{\prime}}}^{{\text{T}}} {\mathbf{e}}_{{\mathbf{z}}} \\ \end{aligned} $$

Note that under the assumption of ‘rigid’ cross sectional planes (no warping), the shear term $\gamma_{yz}$, along with other cross-sectional direct strains will equate to zero. A more detailed discussion of their behaviour, including Poisson’s effects, is highlighted in [3], and is shown to be negligible.

The above developed strain formulations, along with the geometrical relationships from Sect. 3.1, can be used to compile the potential energy of the structure. In the present model, these are generated as Taylor-expanded terms that follows the approach used to establish the geometrical relationships of the problem (e.g., body frame axes). This is done consistently with the aim of retaining up to and including the 3rd order terms in **q** in the final equations of motion. As such, only the terms up to and including the 4th powers of the degrees of freedom are retained in the energy formulations. The applied ordering scheme is further discussed in [30]. The potential energy derived in this manner can be written as,

60 $$ \begin{aligned} {\text{U}} & = \frac{1}{2}\int\limits_{0}^{L} {\iint\limits_{\Pi } {E\varepsilon_{xx}^{2} {\text{d}}A{\text{d}}s}} + \frac{1}{2}\int\limits_{0}^{L} {\iint\limits_{\Pi } {G\left( {\gamma_{xy}^{2} + \gamma_{xz}^{2} } \right){\text{d}}A{\text{d}}s}} \\ & \approx \frac{1}{2}\int\limits_{0}^{L} {E\left( {I_{yy} w^{{\prime\prime}{2}} \left( {1 + w^{{\prime}{2}} } \right) + I_{zz} v^{{\prime\prime}{2}} \left( {1 + v^{{\prime}{2}} } \right)} \right){\text{d}}s} \\ & \quad + \frac{1}{2}\int\limits_{0}^{L} {E\left( {(I_{zz} - I_{yy} )\theta \left( {w^{{\prime\prime}{2}} - v^{{\prime\prime}{2}} } \right) - 2\left( {I_{yy} - I_{zz} } \right)\theta w^{\prime\prime}v^{\prime\prime} + 2I_{zz} v^{\prime}v^{\prime\prime}w^{\prime}w^{\prime\prime}} \right){\text{d}}s} \\ & \quad + \frac{1}{2}\int\limits_{0}^{L} {EI_{yz} \left( {2w^{\prime\prime}v^{\prime\prime} + w^{\prime\prime}v^{\prime\prime}\left( {v^{{\prime}{2}} + w^{{\prime}{2}} } \right) + 2\theta \left( {w^{{\prime\prime}{2}} - v^{{\prime\prime}{2}} } \right) + 2v^{\prime}w^{\prime}w^{{\prime\prime}{2}} - 4\theta^{2} v^{\prime\prime}w^{\prime\prime}} \right)} {\text{d}}s \\  &\quad + \frac{1}{2}\int\limits_{0}^{L} {GJ\left( {\theta^{{\prime}{2}} + v^{{\prime\prime}{2}} w^{{\prime}{2}} + 2\theta^{\prime}v^{\prime\prime}w^{\prime}} \right)} {\text{d}}s \\ \end{aligned} $$

where the terms defining the bending and torsional rigidities for the present model are described in Table 1, along with their numerical values.

Next, the kinetic energy of the structure is developed. The velocity of a generic point on the structure (in the fixed frame *O*) can be written in the form,

61 $$ {\dot{\mathbf{r}}} = {\dot{\mathbf{r}}}_{{{\text{EA}}}} + {{\varvec{\Omega}}} \times \left( {n_{z} {\mathbf{e}}_{{\mathbf{z}}} + n_{y} {\mathbf{e}}_{{\mathbf{y}}} } \right) $$

where ${{\varvec{\Omega}}}({\mathbf{q}},{\dot{\mathbf{q}}})$ describes the angular velocity of the body frame *B* relative to *O*, and is developed in the “Appendix A”. The final form once again originates from the Taylor-expanded expressions with the energy terms only retaining the terms up to and including the 4th order terms. The expression for the kinetic energy is obtained from the following,

62 $$ \begin{aligned} {\text{T}}_{{\text{K}}} & = \frac{1}{2}\int\limits_{0}^{L} {m\left( {{\dot{\mathbf{r}}}_{EA} + {{\varvec{\Omega}}} \times b(a - e){\mathbf{e}}_{y} } \right)^{{\text{T}}} \left( {{\dot{\mathbf{r}}}_{EA} + {{\varvec{\Omega}}} \times b(a - e){\mathbf{e}}_{y} } \right){\text{d}}s} \\ & \qquad + \frac{1}{2}\int\limits_{0}^{L} {m_{xx} \left( {{{\varvec{\Omega}}}^{{\text{T}}} {\mathbf{e}}_{x} } \right)^{2} + m_{zz} \left( {{{\varvec{\Omega}}}^{{\text{T}}} {\mathbf{e}}_{z} } \right)^{2} + m_{yy} \left( {{{\varvec{\Omega}}}^{{\text{T}}} {\mathbf{e}}_{y} } \right)^{2} {\text{d}}s} \\ & \qquad + \frac{1}{2}\sum {m_{s} \left( {{\dot{\mathbf{r}}}_{EA} + {{\varvec{\Omega}}} \times b(a - e_{S} ){\mathbf{e}}_{{\mathbf{y}}} } \right)^{{\text{T}}} \left( {{\dot{\mathbf{r}}}_{EA} + {{\varvec{\Omega}}} \times b(a - e_{S} ){\mathbf{e}}_{{\mathbf{y}}} } \right)} \\ & \qquad + \frac{1}{2}\sum {m_{Sxx} \left( {{{\varvec{\Omega}}}^{{\text{T}}} {\mathbf{e}}_{{\mathbf{x}}} } \right)^{2} + m_{Szz} \left( {{{\varvec{\Omega}}}^{{\text{T}}} {\mathbf{e}}_{{\mathbf{z}}} } \right)^{2} + m_{Syy} \left( {{{\varvec{\Omega}}}^{{\text{T}}} {\mathbf{e}}_{{\mathbf{y}}} } \right)^{2} } \\ \end{aligned} $$

Note that the above expression combines the distributed inertia (through the integrals) and the rigid discrete sources of inertial effects under the summation symbols. The terms defining the inertial and geometrical properties in the above expression are detailed, along with their physical values, in Table 1. It is worth recognising that all the terms resulting from the above are quadratic in the terms of generalised velocities ${\dot{\mathbf{q}}}$, with the remainder of the products in each terms comprising of **q** terms.

## C. Generalised force due to gravity

The formulation for the generalised force that originates due to the weight of the wing is described here. As illustrated in Fig. 2, the fixed frame is placed such that the gravity vector lies in its *y*–*z* plane, with the angle between the gravity vector and the *z* axis being $\alpha_{0}$. The resulting generalised force can be written as,

63 $$ \begin{aligned} {\mathbf{Q}}_{{{\text{grav}}}} & = - \int\limits_{0}^{L} {mg\partial_{{\mathbf{q}}} \left( {{\mathbf{r}}_{{{\text{EA}}}} + b(a - e){\mathbf{e}}_{{\mathbf{y}}} } \right)^{{\text{T}}} \left[ {\begin{array}{*{20}c} 0 \\ {\sin \alpha_{0} } \\ {\cos \alpha_{0} } \\ \end{array} } \right]{\text{d}}s} \\ & \quad - \sum {m_{S} g\partial_{{\mathbf{q}}} \left( {{\mathbf{r}}_{{{\text{EA}}}} + b(a - e_{S} ){\mathbf{e}}_{{\mathbf{y}}} } \right)^{{\text{T}}} \left[ {\begin{array}{*{20}c} 0 \\ {\sin \alpha_{0} } \\ {\cos \alpha_{0} } \\ \end{array} } \right]} \\ \end{aligned} $$

which again is a combination of the distributed inertia (integral) and the discrete inertia (summation). These terms are compiled such that only the terms up to and including 3rd powers of **q** are retained.

## D. Static aerodynamic curves

The data for the static aerodynamic curves used in this work are generated from *xflr* (version 5)^6^ across a range of Reynold’s numbers (100,000 to 300,000) and angles of attack (− 10 to + 10 degrees) for the specific case of the NACA0018 aerofoil. For the case of the lift coefficient and the pitching moment coefficient (about quarter-chord), function in $\alpha$ and *U* are fitted to these data in application to the model. Figure 19 compares the fitted curves against the data for a selected airspeed,

As illustrated by the figure, the lift coefficient appears to exhibit a piecewise linear relationship with $\alpha$. Note that the present analysis is carried out across the pre-stall regime, which with the exemplified speed is reached at approximately 7 degrees.

To account for the piecewise linear behaviour in the lift coefficient, the fitted model is developed in the form of two piecewise linear components, where the gradients in the two regimes being $c_{{l_{\alpha } }}^{I}$ and $c_{{l_{\alpha } }}^{I} + c_{{l_{\alpha } }}^{II}$ respectively.

The fitted static lift coefficient and its gradient are expressed as,

64 $$ \begin{aligned} c_{l} (\alpha ,U) & = c_{{L_{\alpha } }}^{I} (U)\alpha + 0.5\left( {1 + \tanh \left( {50\left( {\left| \alpha \right| - h_{\alpha } (U)} \right)} \right)} \right)c_{{l_{\alpha } }}^{II} (U)\left( {\alpha - {\text{sign(}}\alpha {)}h_{\alpha } (U)} \right) \\ c_{{l_{\alpha } }} (\alpha ,U) & = c_{{L_{\alpha } }}^{I} (U) + 0.5\left( {1 + \tanh \left( {50\left( {\left| \alpha \right| - h_{\alpha } (U)} \right)} \right)} \right)c_{{l_{\alpha } }}^{II} (U) \\ \end{aligned} $$

where,

65 $$ h_{\alpha } (U) = 0.175\left( {0.2139{\text{s/m}} \times U - 1.756} \right) + 0.009 $$

identifies the angle across which the transition between the piece-wise linear regions is noted, and the two gradient functions were fitted as,

66 $$ \begin{aligned} c_{{l_{\alpha } }}^{I} (U) & = \left( {5.975 + 2.3e^{{ - 0.4{\text{s/m}}(U - 10{\text{m/s}})}} } \right) \\ c_{{l_{\alpha } }}^{II} (U) & = 2.675 \\ \end{aligned} $$

The pitching moment about the quarter-chord is fitted as,

67 $$\begin{aligned} c_{m} (U) &= c_{{m_{\alpha } }}^{I} (\alpha ,U)\alpha + 0.5\left( {1 + \tanh \left( {50\left( {\left| \alpha \right| - h_{\alpha } (U)} \right)} \right)} \right)c_{{m_{\alpha } }}^{II} (U)\left( {\alpha - {\text{sign(}}\alpha {)}h_{\alpha } (U)} \right)\\ \end{aligned} $$

where,

68 $$ \begin{aligned} c_{{m_{\alpha } }}^{I} (U) & = 0.2218 - 0.0038\,{\text{s/m}} \times \left( {U - 16.25\,{\text{m/s}}} \right) - 0.25e^{{ - 0.5\,{\text{s/m}} \times (U - 10\,{\text{m/s}})}} \\ c_{{m_{\alpha } }}^{II} (U) & = - 0.675 \\ \end{aligned} $$

The drag coefficient, as opposed to the above is directly interpolated (using the ‘*spline*’ method). Interpolation is done across both the airspeeds (Reynold’s number dependency) and the angle of attack. The data in the two-parameter domain as illustrated in Fig. 20.

## Footnotes

1 Digamma and Polygamma functions in MATLAB: https://uk.mathworks.com/help/matlab/ref/psi.html

2 $\Psi (1 + z) = - \gamma + \sum\nolimits_{n = 1}^{\infty } {\frac{1}{n(z + n)}} = - \gamma + \sum\nolimits_{n = 1}^{\infty } {\left( {\frac{1}{n} + \frac{1}{ - z - n}} \right)}$, for $z \ne - 1, - 2,..$, where $\gamma \approx 0.577$ here is the Euler’s constant.

3 Functions—MATLAB, https://uk.mathworks.com/help/matlab/functions.html Accessed 5th August 2024.

4 Symbolic Computations in MATLAB https://uk.mathworks.com/help/symbolic/symbolic-computations-in-matlab.html Accessed 5th August 2024.

5 Call C/C++ mex functions from MATLAB https://uk.mathworks.com/help/matlab/call-mex-file-functions.html Accessed 5th August 2024.
